# Ammonia Detoxification Inhibits Liver Metastasis by Reshaping Hepatic Microenvironment

**DOI:** 10.1002/advs.202521098

**Published:** 2026-04-03

**Authors:** Sumin Sun, Haili Hu, Long Chen, Yuan Gao, Peng Sun, Liming Chen

**Affiliations:** ^1^ Jiangsu Institute of Cancer Research, Jiangsu Cancer Hospital Affiliated Cancer Hospital of Nanjing Medical University Nanjing Jiangsu China; ^2^ Department of Oncology The Affiliated Huaian No.1 People's Hospital of Nanjing Medical University Huai'an Jiangsu China; ^3^ Department of Biochemistry, School of Life Sciences Nanjing Normal University Nanjing Jiangsu China; ^4^ Jiangsu Key Laboratory of Innovative Cancer Diagnosis & Therapeutics Cancer Institute of Jiangsu Province Nanjing Jiangsu China; ^5^ State Key Laboratory of Oncology in South China Collaborative Innovation Center For Cancer Medicine Guangzhou China; ^6^ Department of Pathology Sun Yat‐sen University Cancer Center Guangzhou China

**Keywords:** ammonia, fibroblasts, liver metastasis, myeloid, tumor microenvironment

## Abstract

Liver metastases represent an urgent unmet medical need in the care of cancer due to a lack of effective therapies. This study reveals that pathological ammonia accumulation, resulting from dysregulation of ammonia removal processes, promotes the formation of a hepatic microenvironment favorable for metastatic colonization. Metabolomic and transcriptomic analyses suggest that tumoral metabolic reprogramming due to cancer cell‐intrinsic hyperactivation of the *de novo* pyrimidine biosynthesis pathway impairs the urea cycle and causes pathological ammonia accumulation. Utilizing tumor microenvironmental profiling and single‐cell RNA sequencing, subsequent analyses further reveal that ammonia accumulation remodels the hepatic stromal‐immune landscape by inducing hepatic stellate cell differentiation into metastasis‐associated fibroblasts (MAFs), suppressing *Ifi27l2a^+^* monocyte‐macrophages, and expanding pro‐metastatic low‐density neutrophils (LDNs). Strikingly, the ammonia detoxifying agent L‐ornithine‐L‐aspartate (LOLA) potently promotes ammonia removal, thereby reshaping the hepatic microenvironment with decrease of MAFs and LDNs and increase of *Ifi27l2a^+^* monocyte‐macrophages, and reducing metastatic burden in liver metastasis mouse models. These results suggest that ammonia, a metabolic toxin, is required for the formation of the hepatic microenvironment favoring liver metastatic colonization, and highlight LOLA as a promising therapeutic strategy for treatment of liver metastasis by detoxifying pathological ammonia accumulation.

## Graphical abstract

Liver metastasis diverts aspartate into hyperactive pyrimidine synthesis, disrupting urea cycling and causing pathogenic ammonia accumulation. Ammonia dually reprograms the microenvironment by: (1) activating hepatic stellate cells (HSCs) into pro‐fibrotic metastasis‐associated fibroblasts (MAFs), and (2) suppressing anti‐tumor monocytes/macrophages (Mono‐AAHM) while expanding pro‐metastatic neutrophils (Neu‐AAHM). L‐ornithine‐L‐aspartate (LOLA) counteracts ammonia toxicity, reduces MAFs, restores anti‐tumor immunity, and suppresses metastasis.

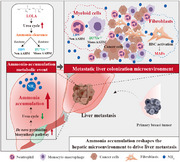

## 1 Introduction

Liver metastases are commonly observed in a spectrum of malignancies, including breast cancer, colorectal cancer, and melanoma [1, 2]. Many malignant tumors remain undiagnosed at early stages, with symptoms typically manifesting only after hepatic dissemination occurs, resulting in markedly low cure rates. The median survival period for untreated liver metastases remains critically limited, ranging from merely 3 to 6 months [3]. Current therapeutic paradigms integrating surgical resection, ablation techniques, and systemic therapies are substantially constrained by tumor heterogeneity, chemoresistance evolution, and the liver's unique immunosuppressive microenvironment [4, 5]. To improve clinical outcomes, it is imperative to decipher the mechanisms underlying liver metastasis progression and develop targeted interventional strategies.

Tumor metabolic reprogramming has been established as a hallmark of cancer [6, 7, 8]. Ammonia has been traditionally considered a toxic byproduct of biomolecular metabolism, including amino acid deamination, and it is well established that the liver is an important metabolic organ in detoxifying ammonia via the urea cycle [9, 10]. Accumulating evidence has shown that tumor cells reintegrate ammonia into biosynthetic pathways as a critical nitrogen source required for tumor cell growth [11]. However, it is largely unknown whether and how hepatic ammonia detoxification function is compromised and whether this is linked to ammonia‐supported tumor colonization in the liver during the development of liver metastasis.

The tumor microenvironment (TME) plays a key role in metastatic colonization, including liver metastasis [12, 13]. Recent studies have reported that ammonia not only serves as a direct nitrogen source for rapid cancer cell proliferation but also plays important roles in shaping a TME that favors tumor cell colonization and growth. For example, ammonia systemically sabotages anti‐tumor immune responses [14, 15]. Despite breakthroughs in understanding ammonia‐mediated adaptive immunoregulation, critical gaps in ammonia‐regulated TME for liver metastasis persist. For example, although ammonia promotes the proliferation of hepatic stellate cells (HSCs) through α‐smooth muscle actin (α‐SMA) activation [16], the role of ammonia in regulating HSC differentiation into metastasis‐associated fibroblasts (MAFs) and in forming metastatic niches during hepatic colonization remains unexplored. While research indicates that metabolic crosstalk (e.g., glucose, lipid, and amino acid metabolism) between myeloid cells and tumor cells directly regulates tumor growth and metastasis [17], systematic studies on the impact of dysregulated ammonia metabolism on myeloid immune cells, including neutrophils and monocyte‐macrophages, during tumor metastasis remain lacking. Thus, further investigation is required to determine the impact of ammonia on the non‐tumor cells, such as HSCs and myeloid cells, in hepatic TMEs, for metastatic colonization of tumor cells in the liver.

In this study, we demonstrate that tumor‐intrinsic pyrimidine synthesis hyperactivation causes urea cycle deficiency in the liver, resulting in hepatic ammonia accumulation that plays important roles in shaping the hepatic TME for metastatic colonization in the liver, including ammonia‐mediated activation of HSCs into MAFs to establish a fibrotic stroma conducive to metastatic growth, and ammonia‐mediated suppression of the antitumor activity of monocyte‐derived macrophages and polarization of neutrophils toward LDN‐like pro‐metastatic subsets. Therapeutic targeting of ammonia metabolism disrupts this self‐reinforcing cycle, markedly attenuating metastatic progression in preclinical liver metastasis mouse models. Our findings position ammonia as a master regulator coordinating metabolic adaptation with multidimensional microenvironmental reprogramming, thereby providing a molecular rationale for combination therapies targeting both metabolic vulnerabilities and immune evasion in liver metastases.

## 2 Results

### 2.1 Targeting *De Novo* Pyrimidine Synthesis Exploits a Metabolic Vulnerability in Liver Metastases

**FIGURE 1:**
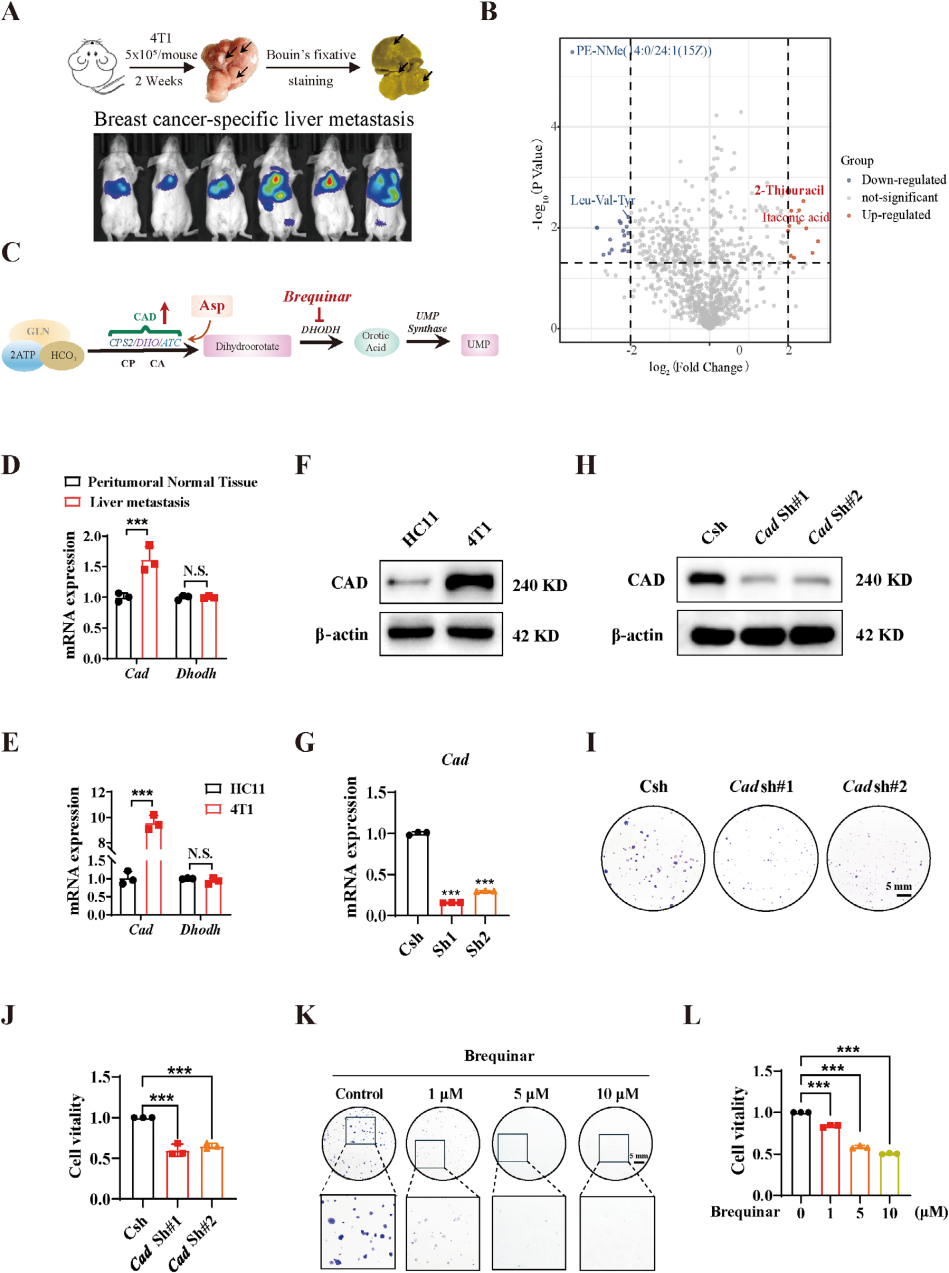
*De novo* pyrimidine synthesis pathway activation in liver metastasis. (A) A schematic diagram with representative images shows that the BCLM model was successfully established in *Balb/c* mice via high‐pressure tail vein injection of highly metastatic 4T1 breast cancer cells. (B) A volcano plot represents the results of untargeted metabolomics of mouse liver metastases versus adjacent non‐tumorous tissues. Significantly downregulated and upregulated metabolites (|log2FC| ≥ 2, p < 0.05) are shown in blue and red, respectively, whereas metabolites without significant changes are shown in gray. 2‐Thiouracil is labeled in bold red text. (C) A schematic diagram of the *de novo* pyrimidine synthesis pathway. (D) RT‐qPCR analysis of pyrimidine synthesis genes *Cad* and *Dhodh* in liver metastasis lesions versus adjacent non‐tumorous tissues in BCLM mice (n = 3). (E) RT‐qPCR analysis of *Cad* and *Dhodh* expression in 4T1 mouse mammary cancer cells compared to normal HC11 mammary epithelial cells (n = 3). (F) Representative Western blot images show elevated CAD protein expression in 4T1 cells compared to HC11 cells. (G, H) Construction and validation of shRNA‐mediated *Cad* knockdown in 4T1 cells (sh*Cad*): (G) RT‐qPCR analysis of *Cad* expression (n = 3); (H) Representative Western blot images showing CAD protein expression. (I, J) Representative images of colony formation and the results of CCK‐8 assays (n = 3) for examining clonogenic capacity and proliferation capability in 4T1 cells with *Cad* knockdown compared to controls. (K, L) Representative images of colony formation and the results of CCK‐8 assays (n = 3) for examining clonogenic capacity and proliferation capability of 4T1 cells treated with the indicated concentrations of Brequinar. The data are expressed as mean ± SD. Statistical analyses were performed using: Student's t‐test (panels D, E) and one‐way ANOVA (G, J, L). ***p < 0.001; N.S., not significant.

Given the liver's central metabolic role, we hypothesized that metastatic tumors activate adaptive pathways through metabolic reprogramming to cope with metabolic stress in the metastatic niche. To test this hypothesis, we established a breast cancer liver metastasis (BCLM) mouse model (Figure 1A). When we performed differential metabolite screening between hepatic metastatic lesions and paired adjacent non‐tumorous tissues using an untargeted metabolomics platform, we found a notable accumulation of 2‐thiouracil in metastatic lesions compared to adjacent non‐tumorous tissues (Figure 1B). This provided evidence of altered pyrimidine metabolism, implying tumor‐intrinsic upregulation of *de novo* pyrimidine synthesis to fulfill nucleotide demands for cancer cell proliferation during metastatic colonization in the liver. Furthermore, analysis of key enzyme expression revealed selective upregulation of the carbamoyl‐phosphate synthetase 2, aspartate transcarbamylase, and dihydroorotase (*Cad*) gene in metastatic lesions and highly invasive 4T1 cells compared to controls (Figure 1C–E; Figure S1). Consistently, elevated CAD protein levels were observed in 4T1 cells compared to HC11 cells (Figure 1F). In contrast, the downstream enzyme dihydroorotate dehydrogenase (*Dhodh*) maintained basal expression (Figure 1C–E). These results suggest that abnormal activation of pyrimidine synthesis is not a general compensatory response but specifically hinges on CAD as the key molecular node.

To define CAD's biological function, we generated *Cad*‐knockdown 4T1 cell lines (sh*Cad*) via lentiviral shRNA delivery for functional assays (Figure 1G, H). The results showed that *Cad* knockdown significantly inhibited tumor clonogenic capacity and cell proliferation (Figure 1I, J), demonstrating that restricted pyrimidine synthesis directly impairs malignant proliferation. To exclude off‐target genetic effects, we pharmacologically inhibited the *de novo* pyrimidine synthesis pathway using Brequinar, a pathway‐specific inhibitor. The results showed that treatment with 1 µM, 5 µM, or 10 µM Brequinar induced a dose‐dependent inhibition of colony formation in 4T1 cells (Figure 1K). Consistent dose‐dependent inhibition was observed in cell viability (Figure 1L). Collectively, our results suggest that liver metastases adapt to the hepatic microenvironment through reprogramming of the *de novo* pyrimidine synthesis pathway, providing a therapeutically targetable liability that furnishes the mechanistic basis for metastasis‐tailored metabolic intervention.

### 2.2 Aspartate Metabolic Reprogramming Drives *De Novo* Pyrimidine Synthesis and Liver Urea Cycle Impairment

**FIGURE 2:**
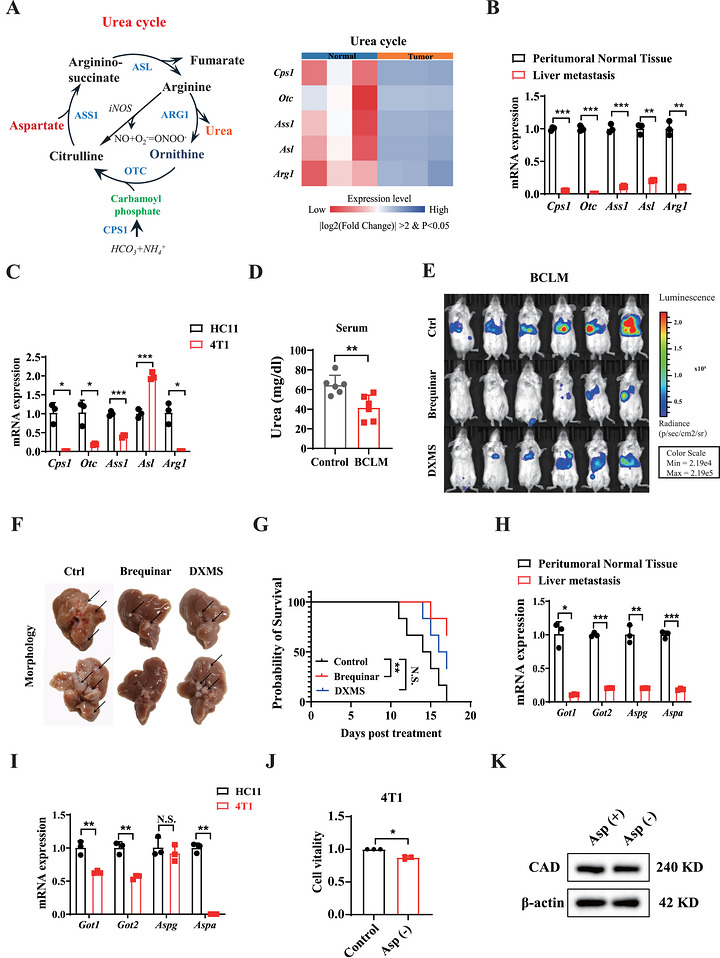
Aspartate metabolic reprogramming drives *de novo* pyrimidine synthesis and liver urea cycle impairment. (A) A schematic diagram of urea cycle (left) and RNA‐seq heatmap showing downregulated expression of urea cycle genes in liver metastases compared with adjacent non‐tumorous tissues in BCLM mice. (B) RT‐qPCR validation of suppressed urea cycle genes (*Cps1*, *Otc*, *Ass1*, *Asl*, *Arg1*) in liver metastases compared to adjacent healthy hepatic tissues in BCLM mice (n=3). (C) RT‐qPCR analysis of the expression levels of urea cycle‐related genes (*Cps1*, *Otc*, *Ass1*, *Asl*, *Arg1*) in 4T1 cells compared to HC11 cells (n = 3). (D) Examination of serum urea in BCLM mice versus healthy controls (n = 6). (E) Representative in vivo bioluminescence images demonstrating suppressed BCLM progression by Brequinar and DXMS compared to controls. (F) Representative morphological images of liver metastases showing differences in tumor nodule size and number among different treatment groups at the experimental endpoint. Selected liver metastases are indicated by black arrows. (G) Survival analysis of BCLM mice treated with Brequinar and DXMS (n = 6). (H) RT‐qPCR analysis of differential Asp biosynthesis gene expression in liver metastatic foci versus adjacent non‐tumorous tissues in BCLM mice (n = 3). (I) RT‐qPCR analysis of Asp biosynthesis gene expression in 4T1 cells compared to HC11 cells (n = 3). (J) The results of CCK8 assay showing 4T1 cell viability in the presence or absence of Asp (n = 3). (K) Representative Western blot images showing the level of CAD protein expression under Asp‐deficient conditions compared to the control. The data are expressed as mean ± SD. Statistical analyses were performed using Student's t‐test (panels B–D, H–J) and Log‐rank test (G). *p < 0.05, **p < 0.01, ***p < 0.001 ; N.S., not significant.

To investigate the regulatory mechanism of *de novo* pyrimidine synthesis in liver metastasis, we performed RNA sequencing (RNA‐seq) analysis. The results revealed a comprehensive deficiency in the expression of genes associated with urea cycle within the metastatic lesions (Figure 2A). This finding was validated by RT‐qPCR experiments (Figure 2B; Figure S2A). These results led us to hypothesize that the downregulation of the urea cycle‐associated genes is linked to pyrimidine metabolic reprogramming. Then, we further investigated the underlying molecular mechanisms. First, in highly metastatic 4T1 cells, we observed significantly suppressed expression of argininosuccinate synthase 1 (*Ass1*), accompanied by markedly decreased expression of key rate‐limiting enzymes in the urea cycle‐carbamoyl phosphate synthetase 1 (*Cps1*), ornithine transcarbamylase (*Otc*), and arginase 1 (*Arg1*) compared to control HC11 cells (Figure 2C). It is worth mentioning that argininosuccinate lyase (*Asl*) expression was unexpectedly elevated in 4T1 cells compared to HC11 cells (Figure 2C). We speculated that this may represent a compensatory attempt within the context of the inherent metabolic defects in tumor cells, primarily driven by the severe limitation in argininosuccinate substrate availability due to the upstream *Ass1* deficiency. However, the increased *Asl* expression failed to reconstitute intact urea cycle functionality in tumor cells. Consistent with this metabolic phenotype displayed by the tumor cells, BCLM mice exhibited a significant reduction in serum urea levels, suggesting impaired overall hepatic urea production capability (Figure 2D). These findings collectively suggest that, during liver metastasis, intrinsic suppression of urea cycle‐related genes (*Ass1*, *Cps1*, *Otc*, *Arg1*) in tumor cells coexists with hyperactivated pyrimidine synthesis.

Critically, in vivo experiments demonstrated that mice treated with the pyrimidine synthesis inhibitor Brequinar or the urea cycle activator Dexamethasone (DXMS) exhibited weaker bioluminescent signals in liver metastases compared to controls (Figure 2E) [18, 19]. This reduction in metastatic burden was corroborated by histopathological examination of liver tissues (Figure 2F). These results suggest that both inhibition of the *de novo* pyrimidine synthesis pathway and activation of the urea cycle can attenuate hepatic metastatic load. Regarding drug safety, no significant differences in body weight were observed among the groups during the treatment period, confirming the absence of apparent systemic toxicity at the experimental doses used (Figure S2B). Subsequent survival analysis revealed significantly prolonged survival periods in Brequinar‐treated mice (Figure 2G). Although not statistically significant, DXMS treatment showed a trend toward extended survival (Figure 2G). We speculate that this discrepancy may stem from the pleiotropic effects of DXMS as a broad‐spectrum glucocorticoid, where its non‐specific activation pathways might compromise the efficacy of targeted urea cycle activation [19]. Collectively, these findings demonstrate that both targeting *de novo* pyrimidine biosynthesis and restoring urea cycle functionality inhibit the malignant progression of BCLM. Further exploration of the crosstalk between these two pathways may hold significant implications for developing BCLM treatment strategies.

This selective impairment within the tumor cells creates a critical metabolic bottleneck specifically at the *Ass1* step. This diversion redirects the shared hub metabolite aspartate (Asp) towards pyrimidine synthesis. Crucially, metastatic lesions and 4T1 cells showed no upregulation of endogenous Asp‐generating enzymes (*Got1/2*, *Aspa*, *Aspg*) (Figure 2H, I; Figure S2C). Furthermore, 4T1 cells exhibited only mild proliferation inhibition under Asp‐restricted conditions, while maintaining stable CAD expression (Figure 2J, K). This confirms that de novo pyrimidine synthesis relies neither on inducing endogenous Asp synthesis enzymes nor on exogenous Asp supply. By downregulating *Ass1*, cancer cells divert Asp away from the urea cycle pathway, repurposing it as fuel for pyrimidine biosynthesis to facilitate proliferation.

Taken together, our data suggest that the autonomy of urea cycle‐related defects within tumor cells underscores the severity of systemic metabolic collapse. Metastatic cells intrinsically compromise the urea cycle through selective enzyme dysregulation (e.g., *Ass1* suppression). Meanwhile, the neighboring non‐cancerous liver parenchyma, despite its vital role in ureagenesis, fails to compensate adequately due to the structural disruption by metastases. These findings not only offer novel perspectives on metabolic remodeling in metastatic malignancies, but also reveal that targeting the pyrimidine‐urea metabolic node may constitute an innovative strategy to overcome cancer‐associated metabolic constraints.

### 2.3 Ammonia Detoxification Inhibits Liver Metastatic Colonization

**FIGURE 3:**
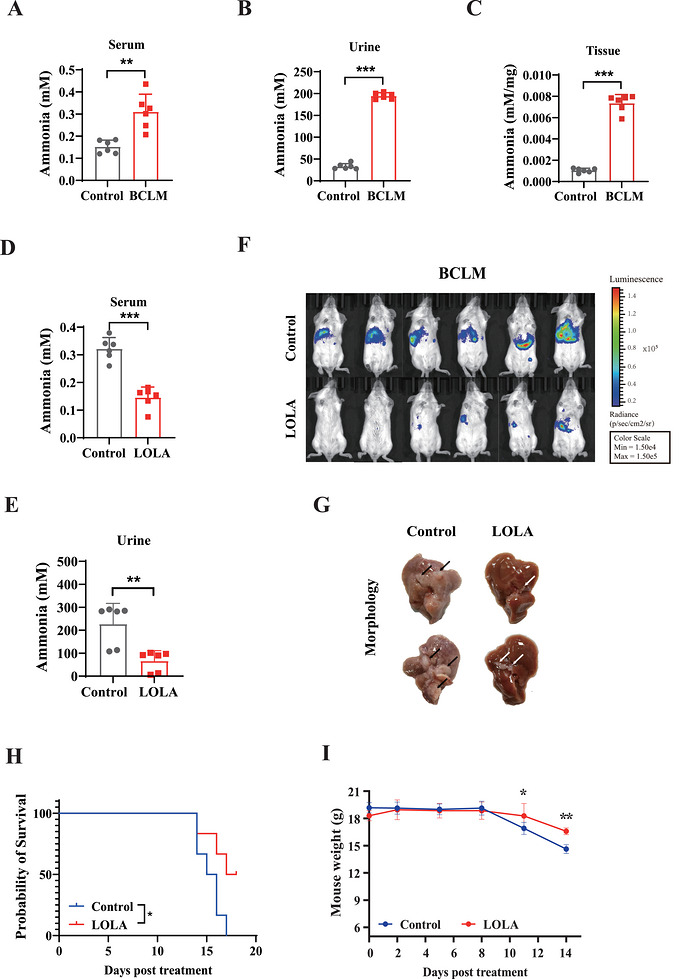
LOLA alleviates hepatic ammonia accumulation and suppresses liver metastasis. (A) Examination of serum ammonia in BCLM mice compared to healthy mouse controls by ammonia assay kit (n = 6). (B) Examination of urinary ammonia in BCLM models compared to healthy mouse controls (n=6). (C) Examination of intratumoral ammonia in liver metastases versus adjacent non‐tumorous tissues in BCLM mice (n = 6). (D) Examination of serum ammonia levels in BCLM mice post‐LOLA intervention compared to solvent controls (n=6). (E) Examination of urinary ammonia in LOLA‐treated BCLM mice compared to solvent controls (n=6). (F) In vivo bioluminescence images of BCLM mice with LOLA treatment compared to solvent controls. (G) Representative photographs show liver gross morphology with multifocal nodular metastases indicated by black arrows in control mice versus reduced metastatic burden indicated by white arrows in LOLA‐treated mice. (H) Overall survival of BCLM mice with LOLA treatment compared to solvent controls (n = 6). (I) Body weight changes in BCLM cohorts with LOLA treatment compared to solvent controls (n=6). The data are expressed as mean ± SD. Statistical analyses were performed using: Student's t‐test (panels A–E); Log‐rank test (H); and two‐way ANOVA (I). *p < 0.05, **p < 0.01, ***p < 0.001.

In murine models of hepatic metastasis, metastatic lesions exhibit significant urea cycle impairment accompanied by pathological accumulation of ammonia. Quantitative assessment of serum, urine, and metastatic liver tissues in BCLM mice revealed substantial ammonia accumulation compared to controls (Figure 3A–C). These results suggest that ammonia as the cytotoxic byproduct accumulates due to urea cycle disruption—primarily driven by preferential substrate allocation toward pyrimidine biosynthesis. These findings led us to hypothesize that dysregulated ammonia metabolism within the hepatic metastatic microenvironment represents not merely a biochemical hallmark of metabolic dysfunction, but potentially a direct driver of tumor progression. To address this issue and investigate the pathological mechanisms underlying ammonia homeostasis imbalance and explore potential intervention strategies, we employed the ammonia‐lowering agent L‐ornithine L‐aspartate (LOLA) in this study. It has been reported that LOLA, which reduces ammonia toxicity by activating the urea cycle, showed significant efficacy in treating hepatic encephalopathy [20]. Our results showed that LOLA treatment significantly reduced systemic ammonia levels in BCLM mice compared to solvent treatment control (Figure 3D, E). These results support urea cycle defects as the primary source of ammonia overload. Furthermore, in vivo, LOLA suppressed hepatic colonization (Figure 3F, G), prolonged survival (Figure 3H), and alleviated body weight loss (Figure 3I). Mechanistically, L‐ornithine enhances mitochondrial ornithine transcarbamylase (OTC) activity to accelerate citrulline synthesis and urea excretion, while L‐aspartate participates in urea cycle replenishment via ASS1‐mediated argininosuccinate formation and transamination‐derived glutamate to activate glutamine synthetase for ammonia conversion. Notably, Asp partitioning exhibits tissue specificity: In intestinal mucosa, Asp generates alanine for hepatic ammonia detoxification and replenishes tricarboxylic acid cycle intermediates, while residual Asp integrates into hepatic mitochondria as a dynamic urea cycle substrate reservoir. Despite potential tumor exploitation of residual Asp for pyrimidine biosynthesis, systemic ammonia reduction conferred dominant antimetastatic benefits, highlighting the pleiotropic advantage of metabolic intervention over pathway‐specific targeting.

To assess the broader applicability of ammonia metabolic targeting, LOLA was evaluated in a colorectal cancer liver metastasis (CCLM) model. The results showed that LOLA treatment markedly attenuated hepatic colonization in CCLM mice (Figure S3A), with histopathology confirming reduced metastatic lesion size and number (Figure S3B). These findings suggest that ammonia dysregulation and urea cycle defects represent convergent features of liver metastatic niches across cancer types. While genomic heterogeneity exists between malignancies, ammonia metabolic imbalance may serve as a pan‐cancer therapeutic vulnerability. Future studies should validate LOLA's efficacy in diverse metastatic models and identify biomarkers (e.g., ASS1 deficiency or hyperammonemia) for patient stratification.

### 2.4 Ammonia Activates MAFs to Promote Pro‐Tumorigenic Fibrotic Microenvironment Formation in the Liver

**FIGURE 4:**
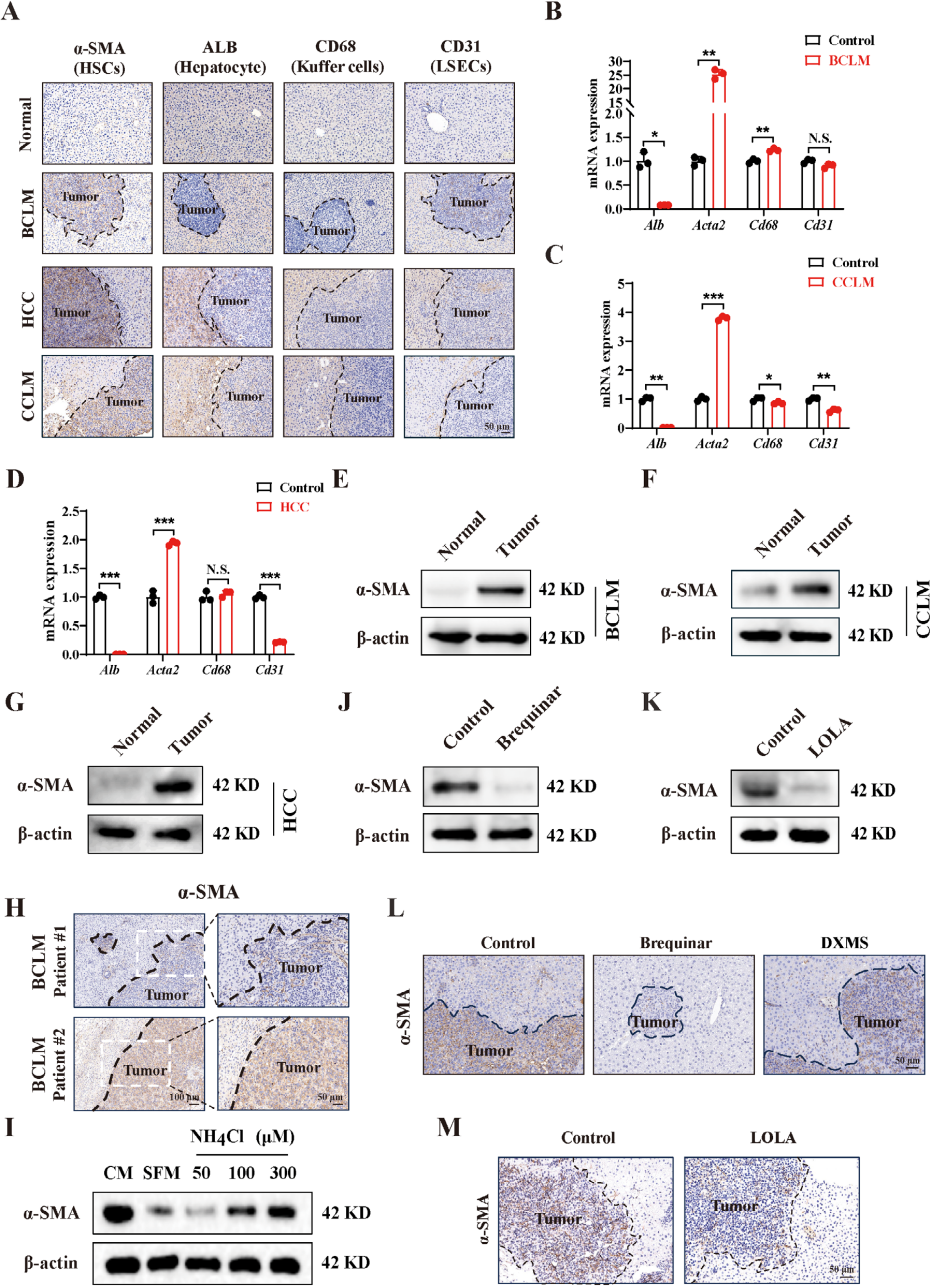
Microenvironmental ammonia imbalance fuels liver metastasis via MAF activation. (A) Representative IHC staining images of liver tissues from healthy mice and BCLM/HCC/CCLM models showing α‐SMA, ALB, CD68, and CD31 distribution in healthy and metastatic liver tissues. Scale bar: 50 µm. (B–D) RT‐qPCR analysis of *Alb*, *Acta2*, *Cd68*, and *Cd31* expression in liver metastatic tumor tissues versus adjacent tissues of BCLM (B), CCLM (C) and HCC (D) mice (n = 3). (E‐G) Representative Western blot images confirming the upregulation of α‐SMA protein in liver metastases compared to adjacent non‐tumorous tissues: (E) BCLM, (F) CCLM and (G) HCC. (H) Representative IHC staining images of human BCLM specimens exhibiting α‐SMA expression patterns in hepatic metastases. Scale bars: 100 µm (left), 50 µm (right). (I) Representative Western blot images showing the α‐SMA protein levels in different concentrations of NH_4_Cl‐treated HSC‐LX2 cells in vitro. (J) Representative Western blot images showing reduced α‐SMA expression in Brequinar‐treated BCLM mice compared to controls. (K) Representative Western blot images showing suppressed α‐SMA levels in LOLA‐treated BCLM mice compared to controls. (L) Representative IHC staining images showing  decreased α‐SMA in livers of BCLM mice treated with Brequinar and DXMS compared to controls. Scale bar: 50 µm. (M) Representative IHC staining images for verification of α‐SMA reduction post‐LOLA treatment in BCLM mice compared to controls. Scale bar: 50 µm. The data are expressed as mean ± SD. Statistical analyses were performed using Student's t‐test. *p < 0.05, **p < 0.01, ***p < 0.001; N.S., not significant.

The liver, as a central metabolic organ, relies on dynamic interactions among diverse cell populations (α‐SMA^+^ HSCs, Albumin^+^/ALB^+^ hepatocytes, CD68^+^ Kupffer cells, and CD31^+^ liver sinusoidal endothelial cells/LSECs) to maintain microenvironmental homeostasis [1]. When we performed immunohistochemical (IHC) staining analyses of BCLM, CCLM, and hepatocellular carcinoma (HCC) models, the results showed a significant expansion of α‐SMA^+^ areas in metastatic lesions compared to normal liver tissues (Figure 4A). It is well‐established that as key effectors in liver tumor microenvironment, HSCs can differentiate into MAFs, thereby forming essential functional units of the pro‐metastatic niche. Additionally, α‐SMA in mouse metastatic tissues was upregulated at both mRNA (*Acta2*) and protein levels (Figure 4B–D and E–G; Figure S4A–C). HSCs are known as a major source of tumor‐associated fibroblasts [21, 22]. These findings suggest that the dynamic increase in α‐SMA expression directly reflects the pathological transition of quiescent HSCs into MAFs, driving the establishment of a pro‐metastasis niche.

Crucially, this phenotypic transformation demonstrates significant clinical relevance. Analysis of liver tissue samples from BCLM patients revealed that metastatic lesions exhibited a significantly higher area of α‐SMA^+^ staining compared to adjacent non‐cancerous regions (Figure 4H). These results indicate that HSC‐to‐MAF transformation represents not only a common response across liver metastasis models but also a key microenvironmental remodeling mechanism in clinical tumor progression. Integrating experimental and clinical data, pathological MAF activation provides essential matrix support and signaling for subsequent metastatic colonization, suggesting its targeting could inhibit microenvironmental malignant transformation.

At the mechanistic level, ammonium chloride (NH_4_Cl)‐induced exogenous ammonia overload dose‐dependently activated HSC‐LX2 cells, driving α‐SMA upregulation (Figure 4I). Furthermore, ammonia exposure promoted excessive deposition of extracellular matrix components (COL1A1, Fibronectin) in activated HSCs, concomitant with MMP9‐mediated matrix remodeling, thereby fostering a profibrotic niche (Figure S4D). These results suggest that ammonia accumulation drives extracellular matrix remodeling, complementing the “ammonia‐liver fibrosis” theory with in vitro mechanistic evidence. To explore the therapeutic potential of targeting the ammonia‐HSCs‐MAFs axis, distinct intervention strategies were employed. Brequinar inhibits *de novo* pyrimidine synthesis, thereby curtailing the indefinite proliferative capacity of tumor cells and subsequently reducing structural damage to normal hepatic tissue. This restores urea cycle function to prevent potentially lethal ammonia accumulation, while DXMS activated the urea cycle enzymes to enhance ammonia clearance, and LOLA directly detoxified ammonia through urea cycle metabolites. Our results showed that although the mechanisms of action of these three strategies were different, all strategies suppressed α‐SMA expression across interventions by reducing microenvironmental ammonia concentration (Figure 4J‐M). This convergence of outcomes establishes ammonia‐MAFs crosstalk as a core regulatory node in liver metastasis.

Taken together, these results suggest that ammonia metabolism imbalance within the liver metastatic microenvironment activates MAFs to induce the formation of a pro‐metastatic stromal niche. Furthermore, multi‐dimensional interventions targeting ammonia metabolism can effectively reverse this pathological process, offering novel insights for stroma‐targeted therapy in liver metastasis.

### 2.5 Blocking MAF Activation Inhibited the Progression of Liver Metastasis

**FIGURE 5:**
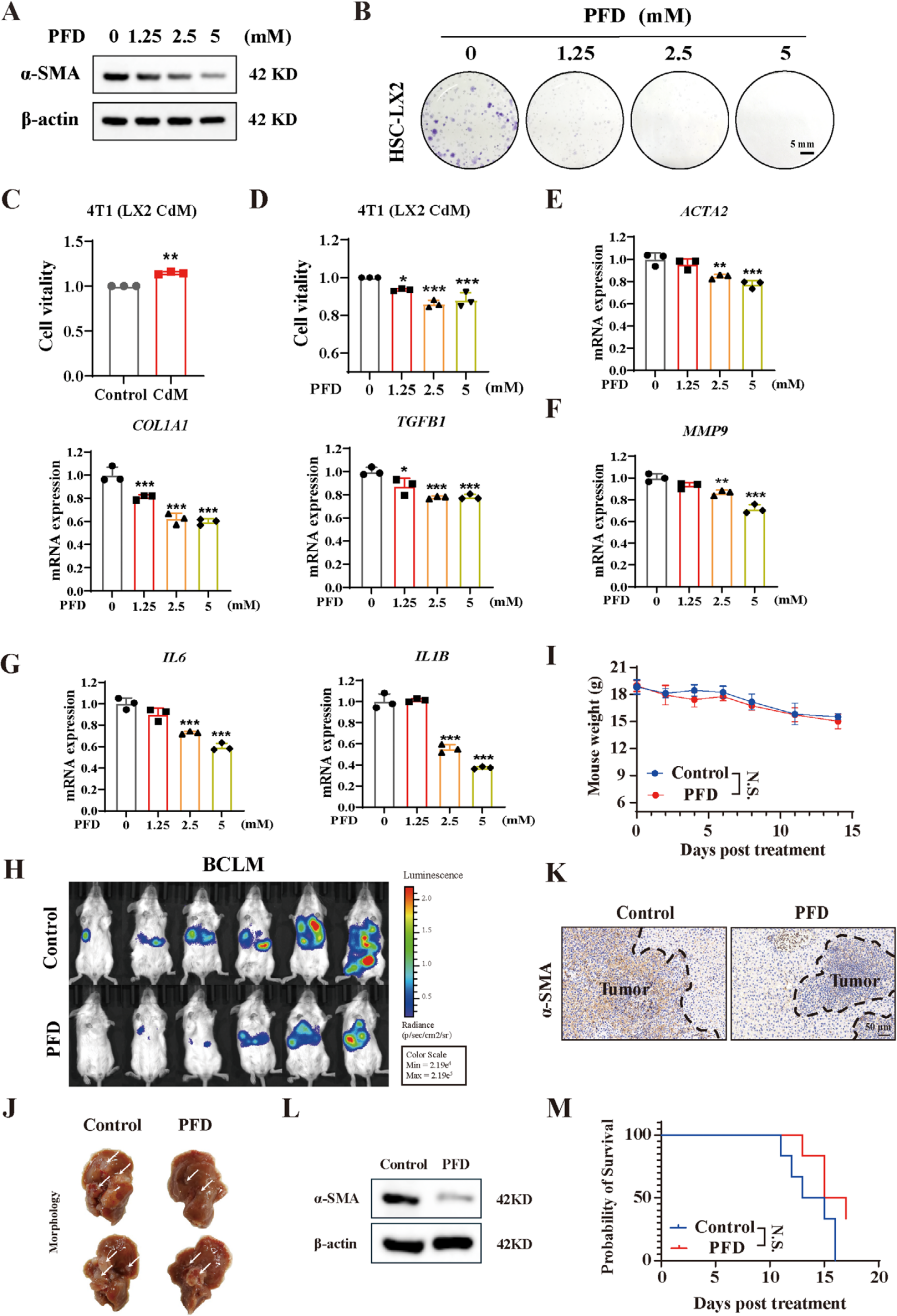
MAF‐targeted intervention suppresses liver metastasis progression. (A) Representative Western blot images showing dose‐dependent α‐SMA suppression in HSC‐LX2 cells (modeling MAF functional status) treated with PFD for 24 h. (B) Representative images of colony formation assay showing the impaired proliferative capacity of MAFs post‐PFD treatment. Scale bar: 5 mm. (C) CCK‐8 assay for measuring cell viability of 4T1 cells exposed to HSC‐LX2‐conditioned medium (CdM) compared to controls (n=3). (D) CCK‐8 assay for measuring the attenuated tumor‐promoting activity of HSC‐LX2‐conditioned medium with PFD (n=3). (E‐G) RT‐qPCR analysis for measuring PFD‐mediated downregulation of: (E) fibrogenic markers (*ACTA2*, *COL1A1*, *TGFB1*), (F) stromal remodeling enzyme (*MMP9*), and (G) inflammatory mediators (*IL6*, *IL1B*) in HSC‐LX2 cells, indicating comprehensive pathway inhibition (n = 3). (H) Representative in vivo bioluminescence images revealing significantly reduced metastatic signals in PFD‐treated livers compared to controls in BCLM model. (I) Body weight changes during PFD treatment compared to controls in BCLM model (n = 6). (J) Representative photos for morphological analysis to demonstrate decreased liver metastasis size and number post‐PFD treatment compared to controls in BCLM mice: selected liver metastases were indicated by arrows. (K, L) Representative images for validation of reduced α‐SMA expression in hepatic metastases from PFD‐treated mice by (K) IHC (Scale bar: 50 µm) and (L) Western blot. (M) Overall survival of mice with indicated treatment (n = 6). The data are expressed as mean ± SD. Statistical analyses were performed using: Student's t‐test (Panel C); one‐way ANOVA (D‐G); two‐way ANOVA (I); and Log‐rank test (M). *p < 0.05, **p < 0.01, ***p < 0.001; N.S., not significant.

Pirfenidone (PFD), an FDA‐approved specific anti‐fibrotic drug, effectively inhibits HSC activation and extracellular matrix deposition by reducing the expression levels of fibrosis markers, including Col1α1, α‐SMA, and TIMP‐1 [23, 24]. It has been reported that PFD can effectively block the liver metastasis process in pancreatic ductal adenocarcinoma by inhibiting HSC activation [25]. Therefore, we hypothesized that PFD may interfere with the formation of a pro‐metastatic microenvironment by suppressing the pathological activation of MAFs. Using an HSC‐LX2 cell model (simulating an activated state) to mimic MAFs functional status, the results showed that PFD treatment dose‐dependently inhibited α‐SMA protein expression and colony formation capacity (Figure 5A, B). Notably, MAFs‐secreted matrix factors significantly enhanced the survival of 4T1 cells, while PFD intervention substantially weakened this pro‐tumor effect (Figure 5C, D). Mechanistic analysis indicated that PFD comprehensively downregulated the core activation genes of MAFs, *ACTA2*, *COL1A1*, and *TGFB1* (Figure 5E) reduced the secretion of the matrix remodeling enzyme *MMP9* (Figure 5F), and concurrently suppressed the expression of inflammatory cytokines *IL6* and *IL1B* (Figure 5G). This suggests that PFD exerts its therapeutic effect by blocking the “pro‐fibrotic‐inflammatory‐matrix degrading” multifunctional axis of MAFs.

Then, we intended to validate the in vitro findings in vivo using our established *Balb/c* mouse BCLM models. The bioluminescence imaging results demonstrated a significantly reduced total hepatic photon flux in PFD‐treated mice compared to controls (Figure 5H). There was no significant difference in body weight changes between the two groups of mice during the treatment period (Figure 5I). Further anatomical assessment confirmed that the PFD group exhibited a reduced number of visible metastatic nodules and a smaller maximum nodule diameter compared to the control group (Figure 5J). Molecular analysis by Western blot and IHC staining showed significant inhibition of pathological MAF activation, manifested as decreased α‐SMA expression in the liver metastases upon PFD treatment compared to controls (Figure 5K, L). However, despite the drug delaying tumor progression, the survival period in the treatment group only showed a non‐significant prolongation trend (Figure 5M). This result suggests that targeting MAFs alone may be insufficient to counteract BCLM progression.

The limitations of monotherapy prompted the future exploration of combination strategies. Previous studies have demonstrated that MAFs induce tumor cells and myeloid cells to co‐upregulate PD‐L1 and other inhibitory molecules by secreting factors such as TGF‐β and IL‐10, thereby establishing a multi‐dimensional immune evasion network [26]. PD‐L1 is an immune checkpoint, and the PD‐1/PD‐L1 signaling axis formed with its receptor PD‐1 is currently one of the most widely recognized and clinically successful targets in cancer immunotherapy [27, 28]. Therefore, this study designed a cross‐dimensional combination intervention strategy and innovatively proposed combining the MAFs activation inhibitor PFD and the ammonia scavenger LOLA, respectively, with a PD‐L1 monoclonal antibody in BCLM mice. The aim was to disrupt the tumor immunosuppressive barrier through multi‐dimensional regulation of matrix/metabolism and immunity. In vivo imaging results showed that monotherapy with the PD‐L1 monoclonal antibody reduced liver bioluminescence to some degree and inhibited the growth of liver metastatic foci, but combining it with PFD did not reveal the anticipated synergistic effect (Figure S5). Furthermore, combining LOLA with PD‐L1 monoclonal antibody treatment paradoxically weakened the anti‐tumor effect (Figure S5). This observation significantly contrasted with the findings of Bell et al. in a colorectal cancer model, whose study confirmed that reducing ammonia toxicity restored T cell function and enhanced the efficacy of PD‐1 blockade [9]. This divergence may be attributed to differences in the genetic background of the disease and organ‐specific microenvironments. Notably, in the breast cancer microenvironment, ornithine supplementation significantly impaired anti‐PD‐1 treatment efficacy and disrupted CD8^+^ T cell function [29], which likely represents the primary reason for the failure of the LOLA and PD‐L1 monoclonal antibody combination.

Taken together, these results suggest that although PD‐L1 monoclonal antibody monotherapy demonstrated a certain anti‐tumor effect in BCLM, the efficacy of combination strategies targeting matrix remodeling (PFD) and metabolic intervention (LOLA) was significantly constrained by tumor genetic background and microenvironmental characteristics. Subsequent research needs to further dissect the metabolic differences between organ metastatic sites and primary tumors while establishing a dynamic ammonia concentration monitoring system to achieve precise control of the therapeutic window. Furthermore, the systematic profiling of tumor microenvironment heterogeneity will serve as an essential scientific foundation for overcoming the bottleneck of combination therapy resistance and realizing precision medicine.

### 2.6 Ammonia Metabolism Modulates the Myeloid Immune Microenvironment in Liver Metastasis

**FIGURE 6:**
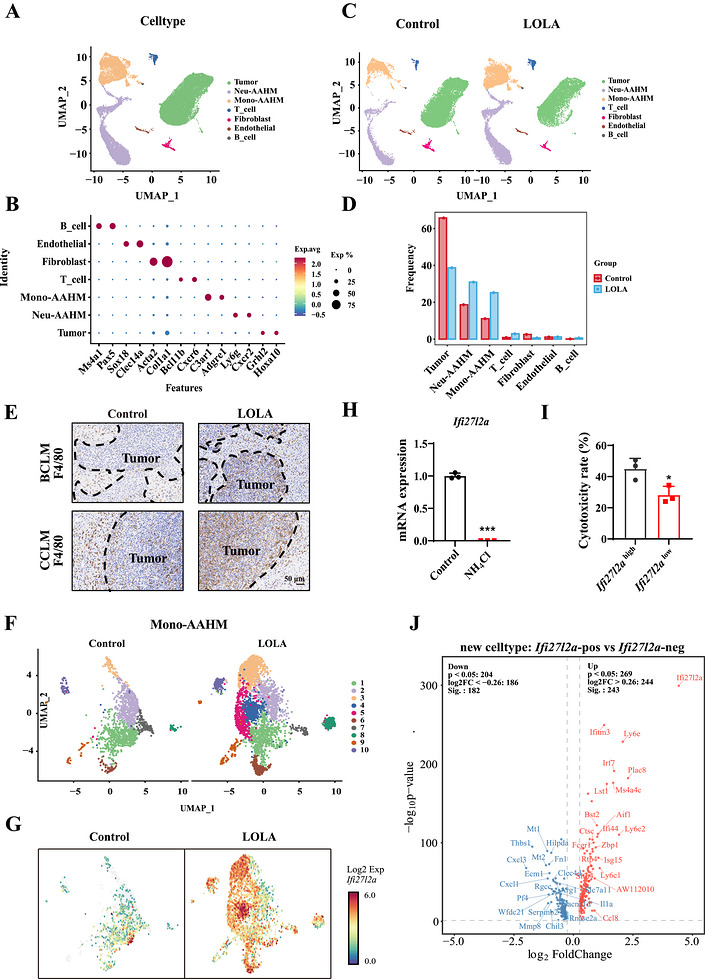
*Ifi27l2a* expression correlates with anti‐tumor function in Mono‐AAHM. (A) UMAP visualization of single‐cell clusters. (B) A feature plot validating cluster annotation through representative cell‐specific marker gene expression. (C) A UMAP plot displaying alterations in cell populations following LOLA intervention. (D) A bar graph depicting proportional changes in cell populations before and after treatment. (E) Representative IHC staining images of F4/80 in BCLM and CCLM models with and without LOLA treatment. Scale bar: 50 µm. (F) A UMAP for comparison of Mono‐AAHM before versus after LOLA administration. (G) *Ifi27l2a* expression localization in Mono‐AAHM UMAP projections. (H) RT‐qPCR analysis of differential *Ifi27l2a* expression in NH_4_Cl‐treated RAW264.7 cells compared to controls (n = 3). (I) CCK‐8 assay for measuring cytotoxic capacity of Mono‐AAHM (*Ifi27l2a* ^low^) and Mono‐AAHM (*Ifi27l2a* ^high^) against 4T1 cells in co‐culture (n = 3). (J) A volcano plot of differentially expressed genes between *Ifi27l2a*‐pos and *Ifi27l2a*‐neg Mono‐AAHM (∣log2FC∣ ≥ 0.26, p < 0.05). The data are expressed as mean ± SD. Statistical analyses were performed using Student's t‐test. *p < 0.05, ***p < 0.001.

To further elucidate the disease mechanisms of liver metastasis, we performed a therapeutic efficacy comparison revealing that mice treated with the ammonia scavenger LOLA exhibited significantly superior survival benefits compared to those receiving PFD (Figure 3H; Figure 5M). This suggests that the therapeutic value of modulating ammonia metabolism for liver metastasis might extend beyond its regulatory effect on MAFs alone. Accordingly, we conducted single‐cell RNA sequencing (scRNA‐seq) analysis on liver metastasis tissues from control and LOLA‐treated mice. Integration of 39,096 single‐cell profiles identified major cell populations, including tumor cells, neutrophil ammonia‐associated hepatic myeloid cells (Neu‐AAHM), mononuclear‐macrophage ammonia‐associated hepatic myeloid cells (Mono‐AAHM), T cells, fibroblasts, endothelial cells, and B cells (Figure 6A), validated by characteristic marker expression (Figure 6B). Critically, quantitative scRNA‐seq analysis showed that LOLA treatment significantly reduced the proportion of tumor cells and fibroblasts, while promoting immune cell infiltration, particularly with the most pronounced changes in ammonia‐associated hepatic myeloid cells (AAHM) (Figure 6C, D). Cell communication analysis further delineated the functional architecture of the niche, revealing that despite their low numerical abundance, fibroblasts acted as dominant signaling hubs, exhibiting the highest interaction frequency and intensity across the entire ecosystem (Figure S6A, B). This stromal centrality positioned them as the primary targets of ammonia‐lowering therapy. Intergroup comparisons demonstrated that LOLA dramatically suppressed pro‐metastatic interactions emanating from fibroblasts to other cell types (Figure S6C, D). Information flow analysis indicated that under LOLA intervention, collagen‐ and extracellular matrix‐related genes (e.g., *Thbs*, *Collagen*, *Fn1*, *Laminin*) were downregulated, while immune‐related genes such as *Mhc‐1*, *Mif*, and *Ccl* were significantly upregulated (Figure S7A). Collectively, these findings demonstrate that lowering ammonia levels via LOLA effectively restructures the metastatic liver microenvironment by suppressing the pro‐fibrotic activity of activated fibroblasts and concomitantly promoting an anti‐tumor immune response.

We further explored the immune microenvironment of liver metastasis with and without LOLA treatment in BCLM mice. The results showed that LOLA markedly increased both the number and frequency of the Mono‐AAHM population compared to controls without LOLA treatment (Figure 6C, D). IHC staining confirmed that LOLA treatment significantly enhanced the infiltration of F4/80^+^ Mono‐AAHM within liver metastatic lesions in both BCLM and CCLM mice compared to controls (Figure 6E). These results suggest that ammonia clearance exerts its anti‐metastatic effects by remodeling the hepatic myeloid immune compartment. Differential gene expression analysis of AAHM identified *Interferon, alpha‐inducible protein 27 like 2A (Ifi27l2a)* as the most significantly enriched gene post‐LOLA treatment compared to the controls (Figure S7B). *Ifi27l2a* expression was upregulated in Mono‐AAHM after LOLA intervention compared to the controls (Figure S7C). Functional annotation revealed that *Ifi27l2a* encodes a mitochondrial‐localized protein previously implicated in driving pro‐inflammatory microglial phenotypes via the ROS‐NR4A axis in stroke models [30]. However, its role in cancer remains unexplored. UMAP analysis demonstrated *Ifi27l2a* enrichment in LOLA‐induced Mono‐AAHM subsets (Figure 6F, G). Furthermore, NH_4_Cl treatment significantly suppressed *Ifi27l2a* mRNA expression in RAW264.7 macrophages compared to the controls (Figure 6H). Based on the marked difference in *Ifi27l2a* expression in macrophages before and after NH_4_Cl treatment, we designated untreated RAW264.7 cells as the *Ifi27l2a* ^high^ group and the NH_4_Cl‐treated group as the *Ifi27l2a* ^low^ group. In co‐culture experiments, *Ifi27l2a* ^high^ macrophages exhibited significantly enhanced tumor‐killing capacity against 4T1 cells compared to *Ifi27l2a* ^low^ groups (Figure 6I). These results suggested that ammonia accumulation impairs Mono‐AAHM anti‐tumor activity in association with *Ifi27l2a* suppression. Thus, *Ifi27l2a* downregulation may serve as a biomarker for Mono‐AAHM dysfunction.

To further investigate the function and mechanism of *Ifi27l2a* in the liver metastasis microenvironment, we segregated Mono‐AAHM cells into *Ifi27l2a*‐positive (*Ifi27l2a*‐pos) and *Ifi27l2a*‐negative (*Ifi27l2a*‐neg) populations. Subsequent scRNA‐seq differential expression profiling combined with GO and KEGG enrichment analyses revealed that the *Ifi27l2a*‐pos population exhibited significant upregulation of pathways including antigen processing and presentation, interferon‐beta response, antiviral defense, proteasome function, and phagosome activity (Figures S8A, S9A). The volcano plot demonstrated upregulation of interferon‐stimulated genes such as *Ifitm3*, *Irf7*, *Bst2*, *Isg15*, and *Zbp1* (Figure 6J). This population was concurrently enriched for MHC class I complex assembly, 2'‐5'‐oligoadenylate synthetase activity, and chemokine function, indicating a state of potent antiviral activity and immune activation (Figure S8A). These findings suggest that *Ifi27l2a* reinforces immune surveillance within the liver metastatic niche by driving interferon signaling and augmenting antigen presentation. Conversely, the *Ifi27l2a*‐neg population displayed characteristics associated with the negative regulation of cell proliferation, activation of IL‐1/IL‐17 and HIF‐1 signaling, resistance to ferroptosis (indicated by upregulation of *Mt1*, *Mt2*, *Slc7a11*, and *Fth1*), and enhanced chemokine signaling activity (Figure 6J; Figures S8B and S9B). This signature suggests a role in mediating immunosuppression and adaptation to metabolic stress. Thus, Ifi27l2a is identified as a pivotal molecule capable of reshaping the immune landscape of liver metastasis. Its positive expression appears to suppress tumor progression by enhancing antiviral‐like response and host antitumor immunity, while its negative expression drives pro‐metastatic inflammatory‐hypoxic networks that feature chemokine signaling enhancement, ferroptosis resistance, and suppressed proliferation.

Taken together, our results suggest that ammonia remodeling of the myeloid immune microenvironment contributes to liver metastasis progression, and suppressed *Ifi27l2a* expression may serve as a potential biomarker for dysfunctional Mono‐AAHM.

### 2.7 Ammonia Metabolism Reprograms Neu‐AAHM Differentiation Trajectory to Orchestrate Anti‐Metastatic Activity

**FIGURE 7:**
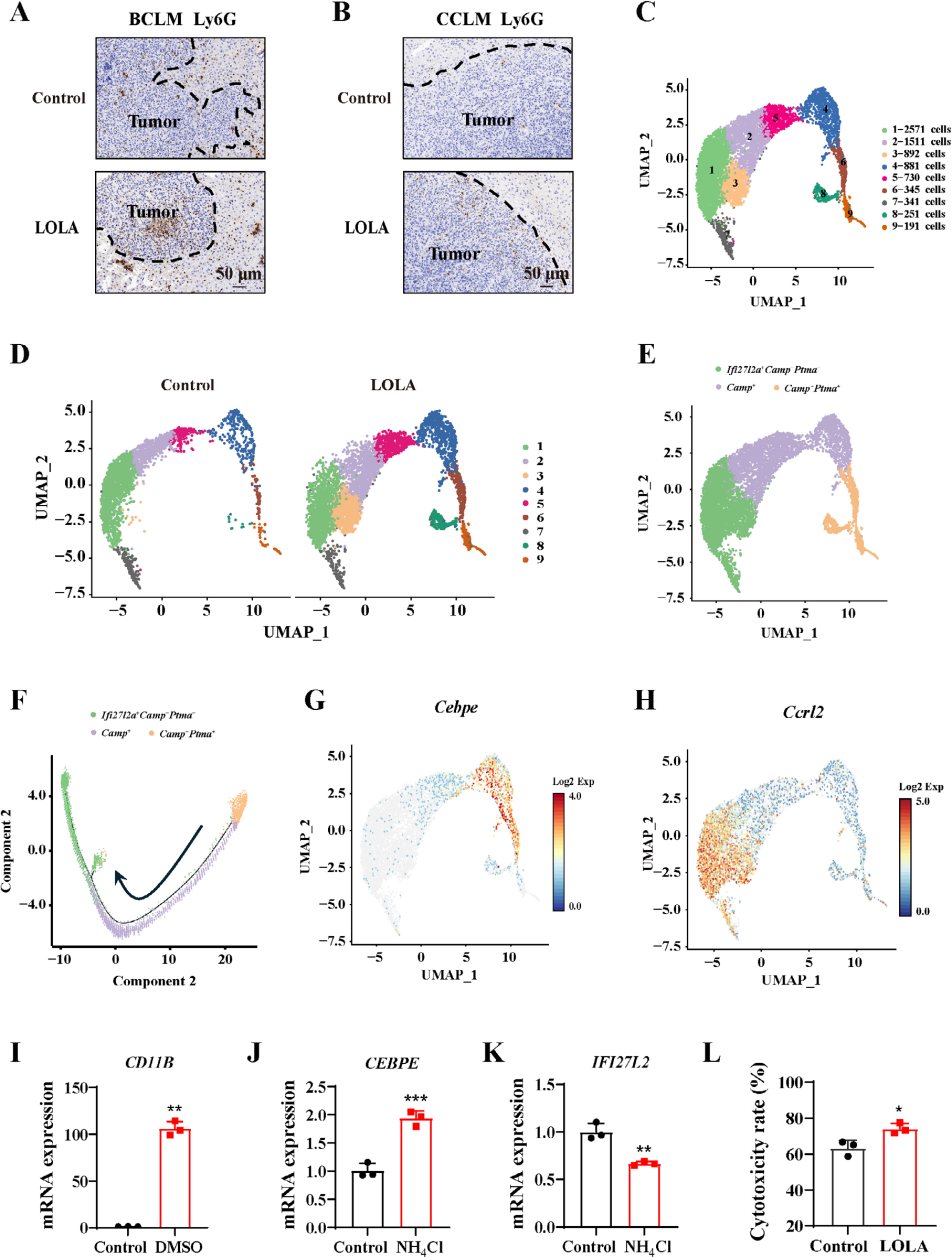
Ammonia accumulation is required for the inhibition of anti‐tumor neutrophils in the TME of mouse liver metastases. (A) Representative IHC images for staining of Ly6G in BCLM model. Scale bar: 50 µm. (B) Representative IHC images for staining of Ly6G in CCLM models. Scale bar: 50 µm. (C) A UMAP clustering map of Neu‐AAHM populations. (D) A Neu‐AAHM UMAP clustering map for analyzing the changes between pre‐ and post‐LOLA intervention. (E) A UMAP map for subclustering analysis of Neu‐AAHM subsets. (F) A pseudotime inference analysis of Neu‐AAHM differentiation trajectories. (G, H) *Cebpe* and *Ccrl2* expression mapping in Neu‐AAHM UMAP space. (I) RT‐qPCR analysis of *CD11B* expression for validation of DMSO‐induced neutrophil differentiation in HL‐60 cells (n = 3). (J) RT‐qPCR analysis of *CEBPE* expression alterations in NH_4_Cl‐treated HL‐60 cells compared to the control (n = 3). (K) RT‐qPCR analysis of *IFI27L2* expression modulation in HL‐60‐derived neutrophils under NH_4_Cl treatment compared to the control (n = 3). (L) In vitro cytotoxic assay using FACS‐sorted LY6G^+^CD11B^+^ neutrophils pre/post‐LOLA treatment (n = 3). The data are expressed as mean ± SD. Statistical analyses were performed using Student's t‐test. *p < 0.05, **p < 0.01, ***p < 0.001.

Our investigation next focused on Neu‐AAHM, another major cell population within the AAHM compartment. IHC analysis of tissue sections from BCLM models revealed that LOLA treatment markedly increased intratumoral Ly6G^+^ Neu‐AAHM in liver metastatic lesions (Figure 7A). In addition, in CCLM models, LOLA treatment also led to a slight but noticeable increase in intratumoral Ly6G^+^ Neu‐AAHM (Figure 7B). These results indicate a crucial role for Neu‐AAHM in suppressing liver metastasis progression. We further found that within the Neu‐AAHM cellular clusters, Neu‐AAHM cells were resolved into nine distinct subsets (Figure 7C). LOLA treatment induced significant structural remodeling, particularly in Cluster 3 (Figure 7D). Using published neutrophil markers, three core subtypes were defined: pro‐tumoral Neu_01_*Camp* ^−^ *Ptma* ^+^ (Clusters 6, 8, 9) [31], anti‐tumoral Neu_02_*Camp* ^+^ (Clusters 2, 4, 5) [31], and self‐identified Neu_03_*Ifi27l2a* ^+^ *Camp^−^ Ptma ^−^* (Clusters 1, 3, 7) (Figure 7E). Pseudotime trajectory analysis revealed Neu‐AAHM differentiation trajectories: Neu_01_ *Camp* ^−^ *Ptma* ^+^ transitions through intermediate Neu_02_*Camp* ^+^ to terminally differentiated Neu_03_*Ifi27l2a* ^+^ *Camp* ^−^ *Ptma^−^* (Figure 7F; Figure S10A). Immature LDN markers (C/EBPε/*Cebpe* and its targets *Hsp90ab1* and *Rpsa*) were enriched in Neu_01 and partially in Neu_02 (Figure 7G; Figure S10B, C), while mature HDN markers (*Ccrl2*, suppressed by C/EBPε) colocalized with Neu_03 (Figure 7H). This transcriptome‐phenotype coupling supports the “pro‐tumor to anti‐tumor” shift during Neu‐AAHM differentiation, with anti‐tumoral Neu_02 and Neu_03 dominating. NH_4_Cl treatment upregulated *CEBPE* in HL‐60‐derived neutrophils (Figure 7I, J). These results suggest that ammonia accumulation drives pro‐tumoral LDN differentiation. Conversely, LOLA‐mediated ammonia clearance suppressed terminal differentiation marker *IFI27L2* in NH_4_Cl‐treated HL‐60 cells (Figure 7K). These results support that ammonia accumulation stalls Neu‐AAHM in pro‐tumoral Neu_01 states. LOLA restored Neu_02/Neu_03 dominance by metabolic reprogramming. Critically, functional validation using flow‐sorted Ly6G^+^CD11B^+^ Neu‐AAHM demonstrated enhanced tumoricidal activity upon LOLA treatment, evidenced by the finding that Neu‐AAHM isolated from LOLA‐treated mice exhibited significantly enhanced cytotoxicity against 4T1 cells in vitro co‐culture assays (Figure 7L). This result provides direct evidence that ammonia clearance reprograms the functional polarization of tumor‐associated neutrophils towards an anti‐tumor effector phenotype.

Collectively, this study reveals an ammonia metabolism‐driven regulatory mechanism governing Neu‐AAHM differentiation. Ammonia accumulation correlates with suppressed *Ifi27l2a* expression and impaired terminal differentiation toward HDN, while LOLA intervention reverses this path through metabolic reprogramming to ultimately enhance anti‐tumor immune surveillance.

## 3 Discussion

This study provides pivotal insights into the mechanisms underlying liver metastasis and identifies novel therapeutic strategies. Firstly, we elucidate the metabolic adaptation pathways enabling malignant cells to establish hepatic niche colonization in the BCLM model. Unlike previous studies emphasizing glucose and lipid metabolism [32, 33], our integrated analysis of metabolomic and transcriptomic profiles revealed a novel competitive metabolic mechanism in liver metastases. Tumor cells systematically hijack Asp resources from the urea cycle to fuel the CAD‐mediated *de novo* pyrimidine synthesis pathway. This prioritization of nucleotide biosynthesis over ammonia detoxification establishes a metabolic trade‐off mechanism, wherein tumor cells sacrifice urea cycle functionality to gain proliferative advantages. Importantly, this mechanistic finding gains crucial translational significance from our preliminary clinical observations. We identified several Triple‐Negative Breast Cancer (TNBC) patients with measurable serum urea levels, a key indicator directly tied to urea cycle activity. Analysis revealed that 25% (2 out of 8) of TNBC patients with liver metastasis exhibited abnormally low serum urea levels (<3.3 mmol/L). Strikingly, this phenomenon was absent in healthy female controls (0 out of 36) and crucially, in a cohort of early‐stage TNBC patients without metastasis (0 out of 3) (Figure S11). This aberrant reduction in serum urea serves as a direct clinical correlate to the compromised urea cycle functionality described in our mechanistic model. Such metabolic rewiring creates a persistent hyperammonemic microenvironment, which may explain the dismal prognosis of liver metastases. Pharmacological interventions targeting pyrimidine synthesis (e.g., Brequinar) or urea cycle reactivation (e.g., LOLA) demonstrated potent reduction of metastatic burden in mice, representing a promising approach to potentially improve outcomes for patients with liver metastases.

Building upon these findings, the study further uncovers ammonia as a master regulator of immunosuppressive microenvironmental remodeling. Functional validation and scRNA‐seq analyses revealed that ammonia accumulation orchestrates multilayered immunosuppression through distinct pathways. A primary mechanism involves the activation of HSCs into α‐SMA^+^ MAFs. The potential mechanism underlying ammonia‐induced HSC‐to‐MAF conversion appears to involve modulation of core signaling pathways, including HIF‐1, IL‐17, TNF, PI3K‐Akt, and MAPK, while concurrently influencing critical biological functions such as extracellular matrix remodeling, hypoxia response, and inflammation (Figure S12A, B). These mechanistic insights currently remain to be validated experimentally; subsequent studies will further elaborate these mechanisms through integrated in vitro and in vivo investigations. Previous studies suggest that dense collagen networks secreted by fibroblasts may form physical barriers restricting T cell infiltration toward the tumor core through mechanical obstruction [34, 35]; additionally, high PD‐L1 expression on the surface of fibroblasts might enhance the functional stability of regulatory T cells via the PD‐1/SHP2 signaling pathway, thereby reinforcing the immunosuppressive microenvironment [36]. These findings challenge the traditional tumor‐centric perspective by emphasizing how metabolic dysregulation cooperates with fibroblasts to establish an immune‐excluded niche. Meanwhile, ammonia accumulation both impairs the anti‐tumor activity of Mono‐AAHM and disrupts neutrophil differentiation by trapping Neu‐AAHM in the LDN state, a process potentially associated with *Ifi27l2a* downregulation. However, the downstream pathways of *Ifi27l2a* and how ammonia regulates the anti‐tumor activity of Mono‐AAHM and drives the differentiation of Neu‐AAHM remain incompletely understood. Future studies will integrate longitudinal sampling across multiple timepoints with scRNA‐seq and spatial transcriptomics to delineate the spatiotemporal heterogeneity of ammonia distribution and its dynamic interplay with distinct immune microenvironments. Critically, LOLA‐mediated ammonia clearance restores Mono‐AAHM‐driven tumor immune surveillance and reverses the C/EBPε‐dependent blockade in neutrophil maturation, underscoring metabolic intervention as a viable strategy to reinstate anti‐tumor immunity. This study constitutes a conceptual advance by establishing the first functional nexus between ammonia metabolism and the myeloid immune axis in liver metastasis, while identifying *Ifi27l2a* as a previously unrecognized master regulator connecting metabolic stress to immune dysregulation—specifically governing Mono‐AAHM anti‐tumor activity and Neu‐AAHM terminal differentiation. Our findings provide an unprecedented paradigm for how metabolic perturbations drive immune reprogramming, with profound mechanistic and therapeutic implications. Furthermore, ammonia modulates Mono‐AAHM immunity via multifaceted mechanisms. Integrative cellular communication analysis revealed prominent interactions between Mono‐AAHMs and CD8^+^ T cells, with signaling pathway dissection highlighting predominant involvement of MHC‐I‐related molecules, providing a mechanistic foundation for future investigation (Figure S13A–C).

Collectively, this work establishes a conceptual framework linking three interconnected axes—pyrimidine‐urea cycle competition, ammonia‐MAFs crosstalk, and ammonia‐*Ifi27l2a*‐AAHM regulation**—**to explain the spatiotemporal dynamics of liver metastasis progression. The identification of *Ifi27l2a* as a bridge between ammonia metabolism and immune regulation offers a potential biomarker for dual‐targeted metabolic‐immunotherapeutic strategies. While these findings provide mechanistic insights, intrinsic species‐specific differences between murine and human liver metastases in immune cell composition, stromal architecture, and disease progression kinetics must be acknowledged. Consequently, the therapeutic concepts derived herein require validation in pathophysiologically representative human models or early clinical investigations before clinical translation. Looking forward, three translational directions warrant prioritization. First, developing ammonia‐responsive nano delivery systems could enhance the spatial precision of metabolic interventions. Second, stratifying patients based on ASS1/CPS1 expression profiles may optimize LOLA responsiveness; however, despite previous reports detailing intravenous 5 g LOLA pharmacokinetics in 10 healthy fasting volunteers and randomized dose‐pharmacokinetic studies in 10 cirrhotic patients [20], the pharmacokinetic signature of LOLA in individuals with liver metastases remains undefined. Third, deciphering the regulatory hierarchy of C/EBPε transcription factors in metabolic‐immune cross‐talk will deepen our mechanistic understanding. These findings collectively advance the paradigm shift from single‐target inhibition to microenvironmental reprogramming, offering a roadmap for precision therapeutics in liver metastasis management.

## 4 Conclusion

In conclusion, our findings delineate an ammonia‐centric pathobiological cascade in BCLM. Tumor‐driven pyrimidine synthesis depletes aspartate and disrupts urea cycling, thereby establishing a pro‐tumorigenic, hyperammonemic niche in the liver. In this niche, ammonia promotes HSC‐to‐MAF differentiation and imposes multilayered immunosuppression via *Ifi27l2a*‐mediated Mono/Neu‐AAHM dysfunction for the metastatic colonization in the liver. Ammonia is a hub regulator of tumor‐stroma‐immune co‐evolution and a promising therapeutic target for BCLM. Future work must validate this axis in diverse liver malignancies to  overcome the current challenges in treating BCLM and other liver metastases.

## 5 Experimental Section

### 5.1 Reagents and Antibodies

L‐Ornithine L‐Aspartate (LOLA) (O7125) and Ammonium Chloride (NH4Cl) (A9434) were acquired from Sigma‐Aldrich (St. Louis, MO, USA). Pirfenidone (PFD) (HY‐B0673) and Brequinar (HY‐108325) were purchased from MedChemExpress (New Jersey, USA). Dulbecco's Modified Essential Medium (DMEM) (PM150210), Roswell Park Memorial Institute 1640 (RPMI 1640) (PM150110) and Iscove's Modified Dulbecco Medium (IMDM) (PM150510) were obtained from Procell (Wuhan, China). Fetal bovine serum (FBS) (086‐150) and trypsin‐EDTA (325‐043‐CL) were supplied by WISENT (Saint‐Jean‐Baptiste, QC, CAN). The primary antibodies against α‐SMA (A17910), CD68 (A13286), and CD31 (A19014) were procured from ABclonal Technology (Wuhan, China). The primary antibody against Carbamoyl Phosphate Synthetase II‐Aspartate Transcarbamylase‐Dihydroorotase (CAD) (93925S) was purchased from Cell Signaling Technology (Danvers, MA, USA). Albumin (16475‐1‐AP) and β‐actin (60008‐1‐Ig) were supplied by Proteintech Group (Wuhan, China). Anti‐CD4 antibody (ab183685), anti‐CD8 antibody (ab217344), anti‐F4/80 antibody (ab300421), and anti‐Ly6G antibody (ab238132) were acquired from Abcam (Cambridge, UK). Anti‐FOXP3 antibody (BS‐0269R) was obtained from Bioss Antibodies Co., Ltd. (Beijing, China).

### 5.2 Animal Procedures and Treatments

All procedures involving animals were reviewed and approved by the Animal Care and Use Committee of Nanjing Normal University (IACUC‐20230912), adhering strictly to the principles outlined in the Guide for the Care and Use of Laboratory Animals. In the BCLM model, female *Balb/c* mice (6‐8 weeks, 18–22 g) were used. For BCLM induction, high‐pressure hydrodynamic tail vein injection of 4T1‐luciferase cells (5×10^5^ cells/1.8 mL PBS, ≤8 s) was performed [37]. In the CCLM model, female *Balb/c* mice (6‐8 weeks, 18–22 g) received high‐pressure hydrodynamic tail vein injection of CT26‐luciferase cells (1.5×10^6^ cells/1.8 mL PBS, ≤8 s). In HCC model, male *Balb/c* mice (6‐8 weeks, 20–22 g) received high‐pressure hydrodynamic tail vein injection of H22 cells (1.5×10^6^ cells/1.8 mL PBS, ≤8 s).

### 5.3 Drug Interventions

(1) Brequinar and DXMS Study: Tumor‐bearing mice were randomly assigned to three groups (n = 6/group): Group 1 (Control) received daily intraperitoneal (i.p.) injections of DMSO/corn oil vehicle (1:9 v/v, 0.1 mL/20 g body weight); Group 2 was administered Brequinar (10 mg/kg in 0.1 mL vehicle, i.p. daily) for 14 days [18]; Group 3 was administered DXMS (10 mg/kg in 0.1 mL vehicle, i.p. every 3 days) for 14 days [38].
(2) PFD Study: Twelve mice were randomized into: Control group receiving oral gavage of vehicle (0.3 mL/20 g daily); and PFD‐treated group administered PFD (300 mg/kg in 0.3 mL vehicle) by daily oral gavage for 14 days [39].
(3) LOLA Study: Twelve mice were divided into: Vehicle control (0.1 mL/20 g oral gavage daily); and LOLA‐treated group receiving LOLA (2 g/kg in 0.1 mL vehicle) via daily oral gavage for 14 days [40].

Upon reaching the experimental endpoint, all animals were euthanized under sodium pentobarbital‐induced anesthesia (50 mg/kg). Liver tissues were immediately dissected and processed through dual pathways: One portion underwent fixation in 4% paraformaldehyde (PFA) followed by paraffin embedding for histopathological assessment, while the remainder was snap‐frozen in liquid nitrogen and stored at −80°C for subsequent protein extraction and biochemical analyses.

### 5.4 Patient Sample Collection

Liver metastasis samples associated with breast cancer were obtained from three patients who underwent surgical resection at Sun Yat‐sen University Cancer Center (G2023‐277‐01). Patients pathologically confirmed with breast cancer and hepatic metastases met the inclusion criteria for this study. All samples and images underwent validation and interpretation by experienced pathologists. This study adhered to the principles of the Declaration of Helsinki and Declaration of Istanbul, and written informed consent was obtained from all participants. The collection of clinical specimens and experimental procedures strictly followed guidelines and protocols approved by the Ethics Committee and relevant committees of Sun Yat‐sen University Cancer Center.

### 5.5 In Vivo Bioluminescence Imaging

Mice were anesthetized with 5% isoflurane/oxygen in an induction chamber and transferred supine to the IVIS Spectrum (PerkinElmer) stage, maintained under 2% isoflurane/oxygen via nose cone. Sterile D‐luciferin potassium salt (150 mg/kg) was injected intraperitoneally; gentle abdominal manipulation was performed to distribute the substrate and minimize leakage. After light shielding and a 10–15 min incubation for bioluminescence signal to stabilize, images were acquired using the IVIS system. Data analysis was performed using Living Image 4.4 software. Post‐imaging, mice recovered individually with activity and food intake monitored for 12 h to ensure complication‐free recovery.

### 5.6 Histological Analysis

Fresh liver specimens were initially fixed in 4% PFA for 12 to 24 h, followed by sequential processing: dehydration through graded ethanol concentrations, clearing in xylene, paraffin embedding, and sectioning. IHC analysis was performed according to established protocols to evaluate the expression of liver metastasis markers [41], including α‐SMA, ALB, CD68, CD31, F4/80, and Ly6G. Representative fields were randomly selected and imaged under bright‐field microscopy in a blinded manner.

### 5.7 Cell Culture

The immortalized cell lines 4T1 (RRID: CVCL_0125), CT26 (RRID: CVCL_7254), and HEK293T (RRID: CVCL_0063) were purchased from the American Type Culture Collection (ATCC, Manassas, VA, USA). Other cell lines, including LX2 (RRID: CVCL_5792), H22 (RRID: CVCL_H613), HL‐60 (RRID: CVCL_0002), RAW264.7 (CVCL_0493) and HC11 (RRID: CVCL_0288) were provided by Procell Sciences Co., Ltd. (Wuhan, China). LX2, RAW264.7 and HEK293T cells were cultured in DMEM supplemented with 10% FBS. 4T1, CT26, H22 and HC11 cells were maintained in RPMI 1640 medium enriched with 10% FBS, while HL‐60 cells were cultured in IMDM supplemented with 20% FBS. All cell lines were authenticated using short tandem repeat DNA profiling by Beijing Tsingke Biotech. Verified cells were generally thawed a few weeks before the experiments and kept in culture less than 6 months. Mycoplasma contamination testing was routinely performed using the PCR Mycoplasma Detection Set (Vazyme, D201‐02), as part of standard procedures in our laboratory's cell bank. All cultures were incubated at 37°C in a humidified atmosphere containing 5% CO_2_ to ensure optimal growth conditions.

### 5.8 In Vitro Drug Treatments

Brequinar treatment in 4T1 cells: Cells were plated in RPMI‐1640 complete medium (10% FBS) and cultured for 24 h (37°C, 5% CO_2_). Subsequent treatments employed graded Brequinar concentrations (1, 5, 10 µM) or vehicle control for 24 h before harvest [42].

PFD treatment in LX2 cells: Cells were seeded in DMEM (10% FBS) for 24 h, followed by exposure to PFD (1.25, 2.5, 5 mM) or vehicle for 24 h [43].

NH_4_Cl‐induced activation of LX2 cells: Cells were seeded in complete medium (CM) supplemented with 10% FBS and cultured at 37°C under 5% CO_2_ for 24 h to facilitate adhesion. Subsequently, the medium was replaced with serum‐free medium (SFM) for an additional 24 h of synchronization. To minimize interference from glutamine metabolism on ammonia cycling, all media were rigorously depleted of exogenous glutamine throughout the experiment. Following synchronization, cells were treated with NH_4_Cl (50, 100, 300 µM) at the indicated concentrations. The NH_4_Cl‐containing medium was renewed every 24 h. After a total treatment period of 72 h, samples were harvested for analysis [16].

### 5.9 Cell Viability Assay

Cell viability was measured using the CCK‐8 assay [44], with absorbance at 450 nm determined using a Bio‐Rad iMark microplate reader.

### 5.10 Lentiviral shRNA Knockdown

Stable 4T1 cell lines expressing CAD shRNA or non‐targeting control shRNA were established using a pLKO.1 lentiviral system. Lentiviral particles, produced by co‐transfecting shRNA constructs (see sequences in Table S1) and packaging plasmids into HEK293T cells, were used to infect target cells. Stable integration was subsequently selected with puromycin [45].

### 5.11 Reverse Transcription Quantitative PCR (RT‐qPCR)

Total RNA isolated from cells was reverse transcribed into cDNA. Quantitative PCR analysis was performed using primers listed in Table S2 with ChamQ SYBR qPCR Master Mix (Vazyme) on a real‐time PCR system, with β‐actin (Actb) as the endogenous control for normalization. Gene expression levels were calculated using the comparative 2^−ΔΔCt^ method.

### 5.12 Western Blot Analysis

Proteins from tissues or cells were extracted as needed. Western blot analysis was performed as described [41], with β‐actin serving as the loading control. Protein expression levels were quantified using ImageJ software from three independent experiments.

### 5.13 Colony Formation Assay

4T1 or LX2 cells (500 cells/well) were seeded in 6‐well plates and treated as required. Following culture until macroscopic colony formation (7–14 days), colonies were fixed with 4% PFA, Giemsa‐stained, PBS‐rinsed, air‐dried, and imaged.

### 5.14 Immunofluorescence Analysis

The immunofluorescence method was adopted to observe the expression and distribution of target protein in HSC‐LX2, and the experimental method was consistent with the previously published article [41]. Fluorescence microscope was used to collect all the images, and representative images were provided.

### 5.15 HL‐60 Differentiation to Neutrophils

HL‐60 cells in the logarithmic growth phase were centrifuged (1000 rpm, 3 min) to remove the original culture medium and resuspended in fresh complete medium. To induce differentiation, DMSO was added to the experimental group medium at a final concentration of 1.25% (v/v). Control cultures received an equivalent volume of sterile PBS. Cells were then continuously cultured at 37°C in a humidified 5% CO_2_ incubator for 5 days. To maintain consistent DMSO concentration, fresh induction medium was replaced every 48 h throughout the culture period. Cell viability was monitored regularly using trypan blue exclusion staining.

### 5.16 Quantitative Determination of Urea and Ammonia Concentrations

Ammonia concentrations in serum, urine, or tumor tissue samples collected from mice were quantified using the Ammonia Assay Kit II (BioAssay Systems, Hayward, CA, USA; SKU D2NH3‐100). Urea levels were determined using the Urea Assay Kit (BioAssay Systems; SKU DIUR‐100). All assays were performed strictly according to the respective manufacturer's protocols.

### 5.17 Untargeted Metabolomics

Twelve mouse tissue samples (6 breast cancer liver metastasis lesions and 6 matched peri‐tumoral normal tissues) were flash‐frozen in liquid nitrogen and stored at −80°C. A 20 mg aliquot was homogenized, followed by addition of 400 µL extraction solvent (methanol:water = 7:3, containing internal standards). The mixture was vortexed (1,500 rpm, 5 min), ice‐bathed for 15 min, and centrifuged (12,000 ×g, 10 min, 4°C). The supernatant (300 µL) was incubated at −20°C for 30 min and centrifuged again (12,000 ×g, 3 min, 4°C). Subsequently, 200 µL of the clarified extract was analyzed by liquid chromatography‐mass spectrometry (LC‐MS). Quality control (QC) pools were included in each batch to monitor technical reproducibility. Chromatographic separation employed an Agilent 1290 LC / 6545 Q‐TOF‐MS system with Waters HSS T3 C18 column (100 × 2.1 mm, 1.8 µm; 40°C) and 0.1% formic acid‐water (A)/acetonitrile (B) gradient: 5–90% B (0–11 min), hold 1 min, re‐equilibration to 5% B. ESI parameters: ±2,500 V/1,500 V capillary voltage, 325°C drying gas, m/z 50–1500 scan range, 135 V fragmentor. Raw data were converted (ProteoWizard), processed via XCMS (peak picking, retention time alignment, SVR normalization), and filtered (>50% group missingness). Metabolites were annotated against HMDB/KEGG/in‐house libraries with MS/MS validation (metDNA algorithm), yielding 3,132 features (1,759 MS/MS‐confirmed). QC stability was verified by total ion chromatogram (TIC) overlay and internal standard CV <15%. Statistical analysis included PCA for clustering, OPLS‐DA for differential metabolite selection (VIP >1), KEGG pathway enrichment (hypergeometric test), and Cytoscape networking. Technical triplicates and 20% standard‐validated metabolites ensured reproducibility. Workflows used R (v3.5.1) with MetaboAnalystR/ComplexHeatmap.

### 5.18 RNA Sequencing (RNA‐seq)

Six mouse tissue samples (3 breast cancer liver metastasis lesions and 3 peri‐tumoral normal tissues) were flash‐frozen in liquid nitrogen and stored at −80°C. Total RNA was extracted using TRIzol Reagent (Invitrogen, #15596026), treated with DNase I to remove genomic DNA, and assessed for purity (NanoDrop A260/A280: 1.8‐2.1) and integrity (1.5% agarose gel; 28S/18S ≥2). The concentration of RNA was accurately measured using the Qubit RNA Broad Range Assay Kit and Qubit 3.0 Fluorometer. Libraries were prepared from 2 µg total RNA with KCTM Stranded mRNA Library Prep Kit, size‐selected (200‐500 bp), and sequenced (150‐bp paired‐end) on MGISEQ‐T7 (triplicate biological replicates). Raw reads were filtered (fastp v0.23.0; adapter/poly‐N/low‐quality removal). Clean reads were aligned to the reference genome, and gene expression was quantified as RPKM. Differentially expressed genes (DEGs) were identified using DESeq2 (P<0.05, |log2FC|>1). Selected DEGs were validated by RT‐qPCR.

### 5.19 Single‐Cell RNA Sequencing (scRNA‐seq)

Fresh BCLM and LOLA‐treated liver metastases from mice were rinsed in ice‐cold PBS, immersed in Miltenyi Tissue Storage Solution (#130‐100‐008; 2–3 pieces/tube, ≈10 mm^3^), and stored at 2–8°C. FASTQ data (10x Genomics) underwent Cell Ranger v9.0.0 processing (GRCm39 alignment) for cell‐gene matrix generation. Seurat v4.0.0 filtered cells (<200 genes, <1,000 UMIs, >5% mitochondrial/erythrocyte genes), removed doublets (DoubletFinder v2.0.3), and normalized data. The top 2,000 HVGs were selected for PCA and UMAP dimensionality reduction. Cluster‐specific markers (Presto‐test) were validated by VlnPlot/FeaturePlot visualization. Differential genes (|FC|>1.5, P<0.05) underwent GO/KEGG enrichment (FDR<0.05). Pseudotime trajectories were inferred via Monocle2 v2.9.0 (ordering genes: q<0.01), validated against CytoTRACE v0.3.3 differentiation scores (0: differentiated; 1: undifferentiated).

### 5.20 In Vitro Cytotoxicity Assay

The cytotoxic activity of RAW264.7 macrophages against 4T1 breast cancer cells was quantified using CCK‐8 assays. Logarithmically growing RAW264.7 and 4T1 cells were prepared and allocated to four experimental groups: (1) Background control (media only) to determine baseline absorbance, (2) Target cell control (4T1 cells alone) to measure baseline proliferation, (3) Effector cell control (RAW264.7 cells alone) to account for phagocyte metabolic activity, and (4) Co‐culture group (RAW264.7:4T1 = 1:1) for cytotoxicity assessment. 4T1 cells (3×10^3^ cells/well) were plated in 96‐well plates with 100 µL medium and allowed to adhere for 6 h. Subsequently, 100 µL medium containing 3×10^3^ RAW264.7 cells was added to co‐culture wells, with control wells receiving fresh medium. After 24 h co‐culture, 20 µL CCK‐8 reagent was introduced per well followed by 1–4 h incubation. Absorbance was measured at 450 nm. Background‐subtracted optical density (OD) values were used to calculate specific cytotoxicity: specific cytotoxicity (%) = [1 – (OD_co‐culture_—OD_effector control_) / OD_target control_] × 100.

### 5.21 Flow Cytometry Assay

Liver metastatic lesions from control and LOLA‐treated mice were dissected, mechanically fragmented by mincing, and subjected to chemical digestion for 90 min at 37°C in dissociation buffer (DMEM containing 0.5 mg/mL collagenase IV, 0.05 mg/mL DNase I, and 5% fetal bovine serum). The resulting cell suspensions were filtered through 70 µm strainers, and isolated cells underwent standard FACS staining procedures. All cells were stained for 30 min at 4°C with fluorescently conjugated anti‐LY6G and anti‐CD11B antibodies in PBS containing 5% FBS to identify neutrophils. Sorted neutrophils were subsequently utilized for in vitro cytotoxicity assays.

### 5.22 Statistical Analysis

Data were expressed as mean ± standard deviation (mean ± SD) and were statistically analyzed using GraphPad Prism 9.0 software. Group differences were assessed using Student's t‐test (normally distributed data) or Mann‐Whitney U test (non‐normally distributed data). For multi‐group comparisons of normally distributed data, one‐way ANOVA followed by Tukey post hoc test (which inherently adjusts for multiple comparisons) was applied. For non‐normally distributed data, Kruskal‐Wallis test with Dunn post hoc testing (incorporating multiplicity correction) was performed. For longitudinal measurements involving two independent factors (e.g., treatment group and time point in body weight tracking), two‐way ANOVA with Šidák's multiple comparisons test was performed to analyze. Survival analysis was performed using the Log‐rank test to assess differences in survival curves between two groups. Statistical significance was defined as p < 0.05 for all analyses.

## Author Contributions

Li.C. supervised the research and is responsible for the overall content as the lead correspondent. S.S. and Li.C. conceived and designed the study. S.S. and Li. C. led the investigation, methodology, validation, and visualization, and wrote the original draft. Li. C. and P.S. contributed to project administration of patient sample and data collection and analysis. Li.C. acquired the funding, led the conceptualization, project administration, and resources, and was responsible for the validation and original draft preparation. S.S. and Li.C. led the writing, review, and editing of the manuscript. H.H., Lo.C. and Y.G. contributed to investigation, methodology, and validation. All authors discussed and commented on the manuscript.

## Conflicts of Interest

Patent applications of this study are filed with Li.C. and S.S. as the inventors, and the authors declare no other competing interests.

## Supporting information




**Supporting File 1**: advs75027‐sup‐0001‐SuppMat.docx.

**Supporting File 2**: advs75027‐sup‐0002‐FigureS1–S13.zip.

## Data Availability

The data that support the findings of this study are available from the corresponding author upon reasonable request.

## References

[1] D. I. Tsilimigras , P. Brodt , P.‐A. Clavien , et al., “Liver Metastases,” Nature Reviews Disease Primers 7, no. 1 (2021): 27, 10.1038/s41572-021-00261-6. 33859205

[2] W. Xu , J. Xu , J. Liu , N. Wang , L. Zhou , and J. Guo , “Liver Metastasis in Cancer: Molecular Mechanisms and Management,” MedComm 6, no. 3 (2020): 70119, 10.1002/mco2.70119. PMC11868442 40027151

[3] L. Zhang , Z. Qiao , Y. Yao , et al., “A Prognostic Model for Triple‐negative Breast Cancer Patients with Liver Metastasis: A Population‐based Study,” Heliyon 10, no. 7 (2024): e27837, 10.1016/j.heliyon.2024.e27837. 38560265 PMC10979062

[4] J. He , Y. Zhang , S. Luo , et al., “Targeting SLC7A11 with sorafenib Sensitizes Stereotactic Body Radiotherapy in Colorectal Cancer Liver Metastasis,” Drug Resistance Updates 81 (2025): 101250, 10.1016/j.drup.2025.101250. 40381225

[5] N. S. Rashid , J. M. Grible , C. V. Clevenger , and J. C. Harrell , “Breast Cancer Liver Metastasis: Current and Future Treatment Approaches,” Clinical & Experimental Metastasis 38, no. 3 (2021): 263–277, 10.1007/s10585-021-10080-4. 33675501 PMC8211035

[6] J. Sun , J. Ding , H. Yue , et al., “Hypoxia‐induced BNIP3 Facilitates the Progression and Metastasis of Uveal Melanoma by Driving Metabolic Reprogramming,” Autophagy 21, no. 1 (2025): 191–209, 10.1080/15548627.2024.2395142. 39265983 PMC11702930

[7] Y. Wang , M. Hu , J. Cao , et al., “ACSL4 and Polyunsaturated Lipids Support Metastatic Extravasation and Colonization,” Cell 188, no. 2 (2025): 412–429.e27, 10.1016/j.cell.2024.10.047. 39591965

[8] A. S. Worral Wilfred Raj and R. Manoharan , “NUAKs Promote mTOR/c‐Myc‐induced Glucose and Glutamine Reprogramming for Cell Growth and Metastasis in Breast Cancer Cells,” Biochimica et Biophysica Acta (BBA)—Molecular Basis of Disease 1871, no. 1 (2025): 167508, 10.1016/j.bbadis.2024.167508. 39270807

[9] H. N. Bell , A. K. Huber , R. Singhal , et al., “Microenvironmental Ammonia Enhances T Cell Exhaustion in Colorectal Cancer,” Cell Metabolism 35, no. 1 (2023): 134–149.e6, 10.1016/j.cmet.2022.11.013. 36528023 PMC9841369

[10] X. Li , H. Zhu , W. Sun , X. Yang , Q. Nie , and X. Fang , “Role of Glutamine and Its Metabolite Ammonia in Crosstalk of Cancer‐associated Fibroblasts and Cancer Cells,” Cancer Cell International 21, no. 1 (2021): 479, 10.1186/s12935-021-02121-5. 34503536 PMC8427881

[11] J. B. Spinelli , H. Yoon , A. E. Ringel , S. Jeanfavre , C. B. Clish , and M. C. Haigis , “Metabolic Recycling of Ammonia via Glutamate Dehydrogenase Supports Breast Cancer Biomass,” Science 358, no. 6365 (2017): 941–946, 10.1126/science.aam9305. 29025995 PMC5748897

[12] S. Zhang , W. Fang , S. Zhou , et al., “Single Cell Transcriptomic Analyses Implicate an Immunosuppressive Tumor Microenvironment in Pancreatic Cancer Liver Metastasis,” Nature Communications 14, no. 1 (2023): 5123, 10.1038/s41467-023-40727-7. PMC10447466 37612267

[13] Y. Liang , J. Li , Y. Yuan , et al., “Exosomal miR‐106a‐5p from Highly Metastatic Colorectal Cancer Cells Drives Liver Metastasis by Inducing Macrophage M2 Polarization in the Tumor Microenvironment,” Journal of Experimental & Clinical Cancer Research 43, no. 1 (2024): 281, 10.1186/s13046-024-03204-7. 39385295 PMC11462797

[14] H. Zhang , J. Liu , W. Yuan , et al., “Ammonia‐induced Lysosomal and Mitochondrial Damage causes Cell Death of Effector CD8^+^ T cells,” Nature Cell Biology 26, no. 11 (2024): 1892–1902, 10.1038/s41556-024-01503-x. 39261719

[15] K. Kurmi and M. C. Haigis , “Nitrogen Metabolism in Cancer and Immunity,” Trends in Cell Biology 30, no. 5 (2020): 408–424, 10.1016/j.tcb.2020.02.005. 32302552 PMC7386658

[16] R. Jalan , F. De Chiara , V. Balasubramaniyan , et al., “Ammonia Produces Pathological Changes in human Hepatic Stellate Cells and Is a Target for Therapy of Portal Hypertension,” Journal of Hepatology 64, no. 4 (2016): 823–833, 10.1016/j.jhep.2015.11.019. 26654994

[17] C. Zhang , Y. Song , H. Yang , and K. Wu , “Myeloid Cells Are Involved in Tumor Immunity, Metastasis and Metabolism in Tumor Microenvironment,” Cell Biology and Toxicology 41, no. 1 (2025): 62, 10.1007/s10565-025-10012-y. 40131539 PMC11937113

[18] Y. Hai , R. Fan , T. Zhao , et al., “A Novel Mitochondria‐targeting DHODH Inhibitor Induces Robust Ferroptosis and Alleviates Immune Suppression,” Pharmacological Research 202 (2024): 107115, 10.1016/j.phrs.2024.107115. 38423231

[19] C. Ulbright and P. J. Snodgrass , “Coordinate Induction of the Urea Cycle Enzymes by Glucagon and Dexamethasone Is Accomplished by Three Different Mechanisms,” Archives of Biochemistry and Biophysics 301, no. 2 (1993): 237–243, 10.1006/abbi.1993.1139. 8460937

[20] G. Kircheis and S. Luth , “Pharmacokinetic and Pharmacodynamic Properties of L‐Ornithine L‐Aspartate (LOLA) in Hepatic Encephalopathy,” Drugs 79, no. 1 (2019): 23–29, 10.1007/s40265-018-1023-2. 30706424 PMC6416235

[21] A. Herrero , E. Knetemann , and I. Mannaerts , “Review: Challenges of in Vitro CAF Modelling in Liver Cancers,” Cancers 13, no. 23 (2021): 5914, 10.3390/cancers13235914. 34885024 PMC8656609

[22] S. Affo , A. Nair , F. Brundu , et al., “Promotion of Cholangiocarcinoma Growth by Diverse Cancer‐associated Fibroblast Subpopulations,” Cancer Cell 39, no. 6 (2021): 866–882.e11, 10.1016/j.ccell.2021.03.012. 33930309 PMC8241235

[23] A. Di Sario , E. Bendia , G. Macarri , et al., “The Anti‐fibrotic Effect of Pirfenidone in Rat Liver Fibrosis is Mediated by Downregulation of Procollagen α1(I), TIMP‐1 and MMP‐2,” Digestive and Liver Disease 36, no. 11 (2004): 744–751, 10.1016/j.dld.2004.05.012. 15571005

[24] Y. Xi , Y. Li , P. Xu , et al., “The Anti‐fibrotic Drug Pirfenidone Inhibits Liver Fibrosis by Targeting the Small Oxidoreductase Glutaredoxin‐1,” Science Advances 7, no. 36 (2021): abg9241, 10.1126/sciadv.abg9241. PMC8442864 34516906

[25] J. Zhao , Y. Zhu , Z. Li , et al., “Pirfenidone‐loaded Exosomes Derived from Pancreatic Ductal Adenocarcinoma Cells Alleviate Fibrosis of Premetastatic Niches to Inhibit Liver Metastasis,” Biomaterials Science 10, no. 22 (2022): 6614–6626, 10.1039/D2BM00770C. 36260512

[26] K. Ganesh , Z. K. Stadler , A. Cercek , et al., “Immunotherapy in Colorectal Cancer: Rationale, Challenges and Potential,” Nature Reviews Gastroenterology & Hepatology 16, no. 6 (2019): 361–375, 10.1038/s41575-019-0126-x. 30886395 PMC7295073

[27] D. Anantha Rajah , H. S. Tan , and R. Farghadani , “Aptamers as Immune Checkpoint Inhibitors in Cancer Immunotherapy: Targeting CTLA‐4/B7 and PD‐1/PD‐L1 Pathways,” International Immunopharmacology 164 (2025): 115339, 10.1016/j.intimp.2025.115339. 40811947

[28] K. Mandal , G. K. Barik , and M. K. Santra , “Overcoming Resistance to Anti‐PD‐L1 Immunotherapy: Mechanisms, Combination Strategies, and Future Directions,” Molecular Cancer 24, no. 1 (2025): 246, 10.1186/s12943-025-02400-z. 41057853 PMC12505684

[29] K. M. Tharp , K. Kersten , O. Maller , et al., “Tumor‐associated macrophages restrict CD8^+^ T cell function Through collagen deposition and metabolic reprogramming of the breast cancer microenvironment,” Nature Cancer 5, no. 7 (2024): 1045–1062, 10.1038/s43018-024-00775-4. 38831058 PMC12204312

[30] G. S. Kim , E. Harmon , M. C. Gutierrez , et al., “Single‐cell Analysis Identifies Ifi27l2a as a Gene Regulator of Microglial Inflammation in the Context of Aging and Stroke in Mice,” Nature Communications 16, no. 1 (2025): 1639, 10.1038/s41467-025-56847-1. PMC11828888 39953063

[31] R. Xue , Q. Zhang , Q. Cao , et al., “Liver Tumour Immune Microenvironment Subtypes and Neutrophil Heterogeneity,” Nature 612, no. 7938 (2022): 141–147, 10.1038/s41586-022-05400-x. 36352227

[32] T. Schild , V. Low , J. Blenis , and A. P. Gomes , “Unique Metabolic Adaptations Dictate Distal Organ‐Specific Metastatic Colonization,” Cancer Cell 33, no. 3 (2018): 347–354, 10.1016/j.ccell.2018.02.001. 29533780 PMC5889305

[33] C. Zhang , X.‐Y. Wang , P. Zhang , et al., “Cancer‐derived Exosomal HSPC111 Promotes Colorectal Cancer Liver Metastasis by Reprogramming Lipid Metabolism in Cancer‐associated Fibroblasts,” Cell Death & Disease 13, no. 1 (2022): 57, 10.1038/s41419-022-04506-4. 35027547 PMC8758774

[34] Y. Chen , J. Kim , S. Yang , et al., “Type I Collagen Deletion in αSMA^+^ Myofibroblasts Augments Immune Suppression and Accelerates Progression of Pancreatic Cancer,” Cancer Cell 39, no. 4 (2021): 548–565.e6, 10.1016/j.ccell.2021.02.007. 33667385 PMC8423173

[35] X. Mao , J. Xu , W. Wang , et al., “Crosstalk between Cancer‐associated Fibroblasts and Immune Cells in the Tumor Microenvironment: New Findings and Future Perspectives,” Molecular Cancer 20, no. 1 (2021): 131, 10.1186/s12943-021-01428-1. 34635121 PMC8504100

[36] M. A. Lakins , E. Ghorani , H. Munir , C. P. Martins , and J. D. Shields , “Cancer‐associated Fibroblasts Induce Antigen‐specific Deletion of CD8^+^ T Cells to Protect Tumour Cells,” Nature Communications 9, no. 1 (2018): 948, 10.1038/s41467-018-03347-0. PMC5838096 29507342

[37] J. Li , Q. Yao , and D. Liu , “Hydrodynamic Cell Delivery for Simultaneous Establishment of Tumor Growth in Mouse Lung, Liver and Kidney,” Cancer Biology & Therapy 12, no. 8 (2011): 737–741, 10.4161/cbt.12.8.16442. 21832881 PMC3218527

[38] A. Dinarello , T. S. Mills , I. W. Tengesdal , N. E. Powers , T. Azam , and C. A. Dinarello , “Dexamethasone and OLT1177 Cooperate in the Reduction of Melanoma Growth by Inhibiting STAT3 Functions,” Cells 12, no. 2 (2023): 294, 10.3390/cells12020294. 36672229 PMC9856388

[39] M. Inomata , K. Kamio , A. Azuma , et al., “Pirfenidone Inhibits Fibrocyte Accumulation in the Lungs in Bleomycin‐induced Murine Pulmonary Fibrosis,” Respiratory Research 15, no. 1 (2014): 16, 10.1186/1465-9921-15-16. 24507087 PMC3930125

[40] A. Najmi , K. Pillai , S. Pal , M. Akhtar , M. Aqil , and M. Sharma , “Effect of L‐ornithine L‐aspartate against Thioacetamide‐induced Hepatic Damage in Rats,” Indian Journal of Pharmacology 42, no. 6 (2010): 384–387, 10.4103/0253-7613.71926. 21189911 PMC2991698

[41] S. Sun , Z. Li , S. Huan , et al., “Modification of Lysine Deacetylation Regulates Curcumol‐induced Necroptosis Through Autophagy in Hepatic Stellate Cells,” Phytotherapy Research 36, no. 6 (2022): 2660–2676, 10.1002/ptr.7483. 35545249

[42] D. C. Schultz , R. M. Johnson , K. Ayyanathan , et al., “Pyrimidine Inhibitors Synergize with Nucleoside Analogues to Block SARS‐CoV‐2,” Nature 604, no. 7904 (2022): 134–140, 10.1038/s41586-022-04482-x. 35130559 PMC10377386

[43] Y. Yang , Y. Ye , X. Lin , K. Wu , and M. Yu , “Inhibition of Pirfenidone on TGF‐beta2 Induced Proliferation, Migration and Epithlial‐mesenchymal Transition of human Lens Epithelial Cells Line SRA01/04,” PLoS ONE 8, no. 2 (2013): 56837, 10.1371/journal.pone.0056837. PMC3578851 23437252

[44] S. Sun , H. Hu , F. Li , et al., “Salidroside Enhances 5‐Fluorouracil Sensitivity against Hepatocellular Carcinoma via YIPF5‐induced Mitophagy,” Frontiers in Pharmacology 15 (2024): 1503490, 10.3389/fphar.2024.1503490. 39834805 PMC11743563

[45] L. Huang , J. Zhang , B. Wei , et al., “Small‐molecule MHC‐II Inducers Promote Immune Detection and Anti‐cancer Immunity via Editing Cancer Metabolism,” Cell Chemical Biology 30, no. 9 (2023): 1076–1089.e11, 10.1016/j.chembiol.2023.05.003. 37236192

