# Supplementary material for: Ammonia Detoxification Inhibits Liver Metastasis by Reshaping Hepatic Microenvironment
Source: Adv Sci (Weinh). 2026 Apr 3;13(33):e21098. doi: 10.1002/advs.202521098 (PMC13271603; doi:10.1002/advs.202521098)
Supplement: Supplementary file 1 — Supporting File 1: advs75027‐sup‐0001‐SuppMat.docx. [file ADVS-13-e21098-s002.docx]

Supporting Information

Title

**Ammonia Detoxification Inhibits Liver Metastasis by Reshaping Hepatic Microenvironment**

*Sumin Sun, Haili Hu, Long Chen, Yuan Gao, Peng Sun* and Liming Chen**

**Figure S1.** (A) RT-qPCR analysis of pyrimidine synthesis genes *Cad* in BCLM lesions and the liver tissues of healthy mice (n = 3). The data are expressed as mean ± SD. Statistical analyses were performed using Student's t-test. **p < 0.01 vs. Normal liver tissues.

**Figure S2.** (A) RT-qPCR validation of urea cycle genes (*Cps1*, *Otc*, *Ass1*, *Asl*, *Arg1*) in BCLM lesions and the liver tissues of healthy mice (n = 3). (B) Treated mice showed no significant weight loss compared with controls, indicating that the drug was well-tolerated at the experimental dose (n = 6). (C) Differential Asp biosynthesis gene expression in metastatic foci versus healthy mice liver tissues (n = 3). The data are expressed as mean ± SD. Statistical analyses were performed using Student's t-test (A, C) and two-way ANOVA (B). *p < 0.05, **p < 0.01, ***p < 0.001 vs. Control. N.S., not significant.

**Figure S3.** (A) In CT26-luc cells established CCLM models, LOLA treatment reduced hepatic bioluminescence intensity. (B) Representative images showing decreased metastatic nodules in LOLA-treated CCLM livers.

**Figure S4.** (A) RT-qPCR analysis comparing *Acta2* in metastatic foci versus healthy mice of BCLM (n = 3). (B) RT-qPCR analysis comparing *Acta2* in metastatic foci versus healthy mice of CCLM (n = 3). (C) RT-qPCR analysis comparing *Acta2* in metastatic foci versus healthy mice of HCC (n = 3). (D) Immunofluorescence analysis of COL1A1, fibronectin, and MMP9 expression in HSC-LX2 cells treated with 300 μM NH₄Cl. The data are expressed as mean ± SD. Statistical analyses were performed using Student's t-test. *p < 0.05, ***p < 0.001 vs. Normal liver tissues.

**Figure S5.** (A) *In vivo* imaging evaluation of the therapeutic efficacy of PD-L1 monoclonal antibody combined with PFD and LOLA.

**Figure S6.** (A, B) Cell-cell communication network analysis: interaction numbers and strength across clusters. (C, D) Changes in intercellular communication numbers and strength between clusters after LOLA administration.

**Figure S7.** (A) Alterations in cellular information flow pre/post-LOLA treatment via single-cell analysis. (B) Differentially expressed genes in AAHM population before/after LOLA dosing. (C) Violin plot depicting *Ifi27l2a* expression dynamics in Mono-AAHM after LOLA treatment.

**Figure S8.** (A, B) GO analysis of *Ifi27l2a*-pos and *Ifi27l2a*-neg Mono-AAHM.

**Figure S9.** (A, B) KEGG analysis of *Ifi27l2a*-pos and *Ifi27l2a*-neg Mono-AAHM.

**Figure S10.** (A) CytoTRACE analysis of Neu-AAHM. (B, C) UMAP visualization of gene expression in Neu-AAHM: (B) *Hsp90ab1*, (C) *Rpsa*.

**Figure 11.** Proportions of low urea cycle metabolism in healthy females, non-metastatic TNBC patients, and TNBC patients with liver metastases.

**Figure S12.** (A) GO analysis of fibroblasts in the control group and the LOLA group. (B) KEGG analysis of fibroblasts in the control group and the LOLA group.

**Figure S13.** (A, B) Cell-cell communication analysis between Mono-AAHM and T-cell subsets. (C) Bubble plot of significant signaling pathways in Mono-AAHM/T-cell crosstalk.

**Table 1 shRNA sequences**

| shRNA | Sequence (5'-3') |
| --- | --- |
| *Cad* shRNA#1 | GCCTCCACGAAAGGTTCTAAT |
| *Cad* shRNA#2 | CCCTTCCTACAAAGCTCAGAT |

**Table 2 RT-qPCR primer sequences**

| Primer name | Sequence (5'-3') |
| --- | --- |
| *Cad* qF | AACTCTGGAGCGGATCAAAGCC |
| *Cad* qR | GGAAAGAGCGAGATACACGCAC |
| *Dhodh* qF | TCTTCACCTCTTACCTGACAGC |
| *Dhodh* qR | CATGTTGGAGTCCTGAAACGTA |
| *Cps1* qF | CATGGAACATCCAGCCGAATTGG |
| *Cps1* qR | GATGGCACATCCTCAGAGCCTT |
| *Otc* qF | ACACTGTTTGCCTAGAAAGCCAG |
| *Otc* qR | CTTCTGGAGCACAGGTGAGTAG |
| *Ass1* qF | CACTCTACGAGGACCGCTATCT |
| *Ass1* qR | CTCAAAGCGGACCTGGTCATTC |
| *Asl* qF | GGCAGAGACTAAAGGAGTGGCT |
| *Asl* qR | TCGACACTGGATTTCGCTGTGC |
| *Arg1* qF | CATTGGCTTGCGAGACGTAGAC |
| *Arg1* qR | GCTGAAGGTCTCTTCCATCACC |
| *Got1* qF | TGCTACTGGGATGCGGAGAAGA |
| *Got1* qR | TGCATGACAGCAGCGATCTGCT |
| *Got2* qF | GCGGTTTTGACTTCTCTGGAGC |
| *Got2* qR | ACGGACGCTATCTCCTTCCACT |
| *Aspg* qF | CTGACCAGAGGATCATCTACACG |
| *Aspg* qR | CTCTATGGTTTGGGCAATCTGAA |
| *Aspa* qF | ACATGGCTGCTGTTATTCATCC |
| *Aspa* qR | GGGTACACGGTACAGTCTCCA |
| *Slc25a13* qF | TGGCAACAGGAAAGACGTGGAG |
| *Slc25a13* qR | CCGCTCAATGTCTGCTAAGGTC |
| *Alb* qF | TGCTTTTTCCAGGGGTGTGTT |
| *Alb* qR | TTACTTCCTGCACTAATTTGGCA |
| *Acta2* qF | TGCTGACAGAGGCACCACTGAA |
| *Acta2* qR | CAGTTGTACGTCCAGAGGCATAG |
| *Cd68* qF | GGCGGTGGAATACAATGTGTCC |
| *Cd68* qR | AGCAGGTCAAGGTGAACAGCTG |
| *Cd31* qF | CCAAAGCCAGTAGCATCATGGTC |
| *Cd31* qR | GGATGGTGAAGTTGGCTACAGG |
| *ACTA2* qF | CTATGCCTCTGGACGCACAACT |
| *ACTA2* qR | CAGATCCAGACGCATGATGGCA |
| *COL1A1* qF | GATTCCCTGGACCTAAAGGTGC |
| *COL1A1* qR | AGCCTCTCCATCTTTGCCAGCA |
| *TGFB1* qF | TACCTGAACCCGTGTTGCTCTC |
| *TGFB1* qR | GTTGCTGAGGTATCGCCAGGAA |
| *MMP9* qF | GCCACTACTGTGCCTTTGAGTC |
| *MMP9* qR | CCCTCAGAGAATCGCCAGTACT |
| *IL1B* qF | CCACAGACCTTCCAGGAGAATG |
| *IL1B* qR | GTGCAGTTCAGTGATCGTACAGG |
| *IL6* qF | AGACAGCCACTCACCTCTTCAG |
| *IL6* qR | TTCTGCCAGTGCCTCTTTGCTG |
| *Ifi27l2a* qF | CTTCACTGGGACAGGCATTGCA |
| *Ifi27l2a* qR | CCTGCTGATTGGAGTGTGGCTA |
| *CD11B* qF | GGAACGCCATTGTCTGCTTTCG |
| *CD11B* qR | ATGCTGAGGTCATCCTGGCAGA |
| *IFI27L2* qF | ATGATGTCCGCAGCAGCCATTG |
| *IFI27L2* qR | CCAACACTGACCCAACAGAGGC |
| *CEBPE* qF | CCAGCCTCTGCGCGTTCTCAA |
| *CEBPE* qR | CAAGGCTATCTTTGTTCACTGCC |
