# Supplementary figures and images for: Ammonia Detoxification Inhibits Liver Metastasis by Reshaping Hepatic Microenvironment
Source: Adv Sci (Weinh). 2026 Apr 3;13(33):e21098. doi: 10.1002/advs.202521098 (PMC13271603; doi:10.1002/advs.202521098)

**Figure S1**

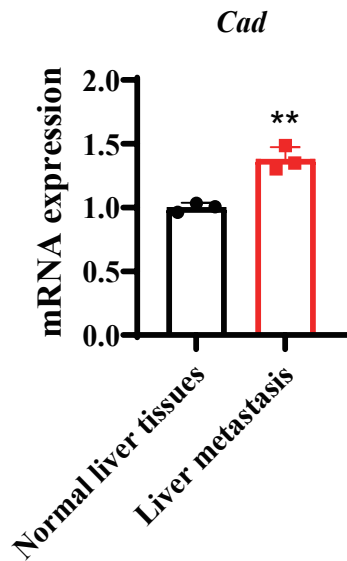

Supplement: Supplementary file 2 — Supporting File 2: advs75027‐sup‐0002‐FigureS1–S13.zip. [file ADVS-13-e21098-s001.zip › Figure S1.pdf]

Figure S10

A

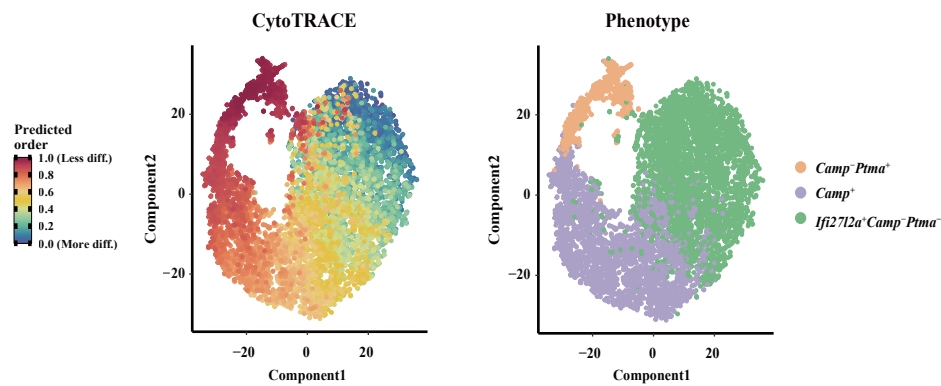

B

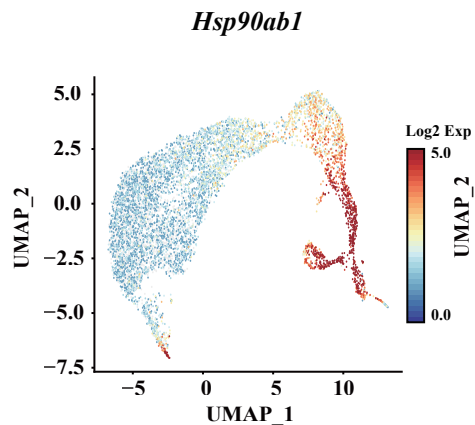

C

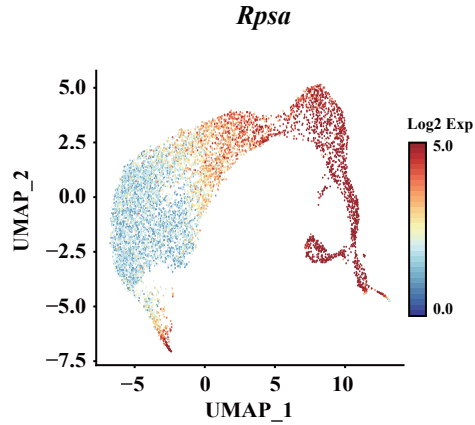

Supplement: Supplementary file 2 — Supporting File 2: advs75027‐sup‐0002‐FigureS1–S13.zip. [file ADVS-13-e21098-s001.zip › Figure S10.pdf]

**Figure S11**

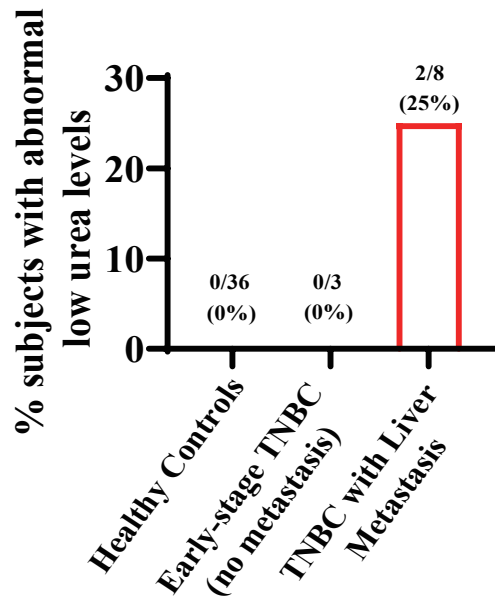

Supplement: Supplementary file 2 — Supporting File 2: advs75027‐sup‐0002‐FigureS1–S13.zip. [file ADVS-13-e21098-s001.zip › Figure S11.pdf]

Figure S12

A

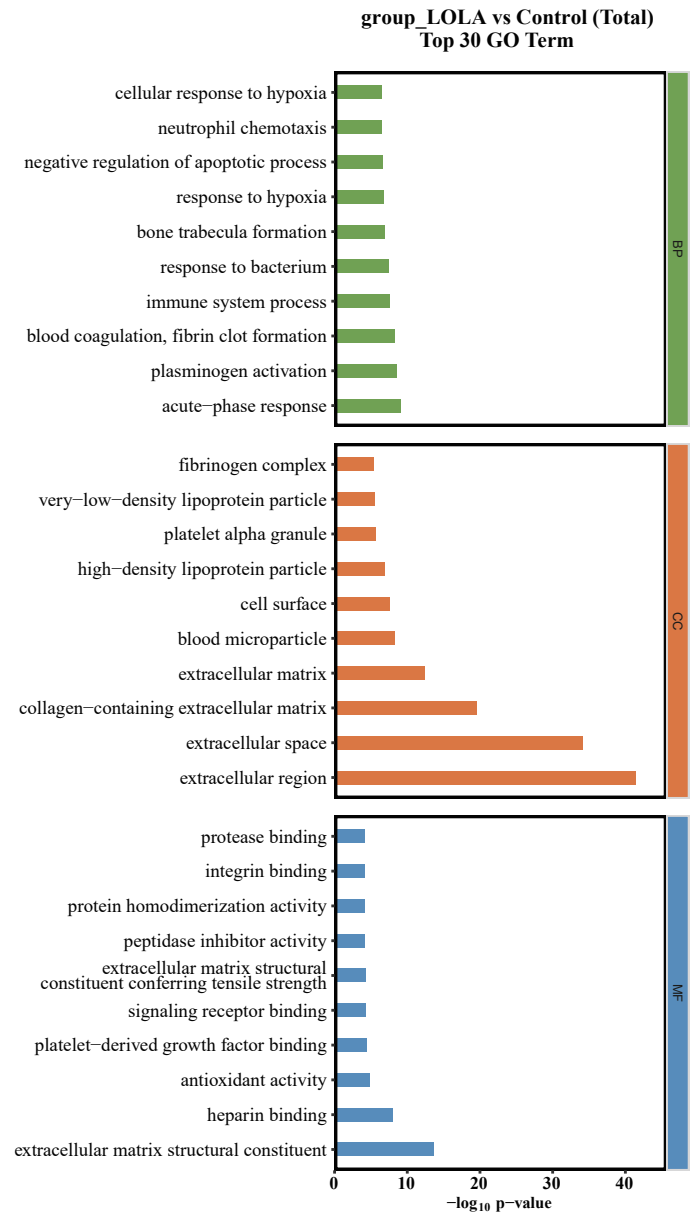

B

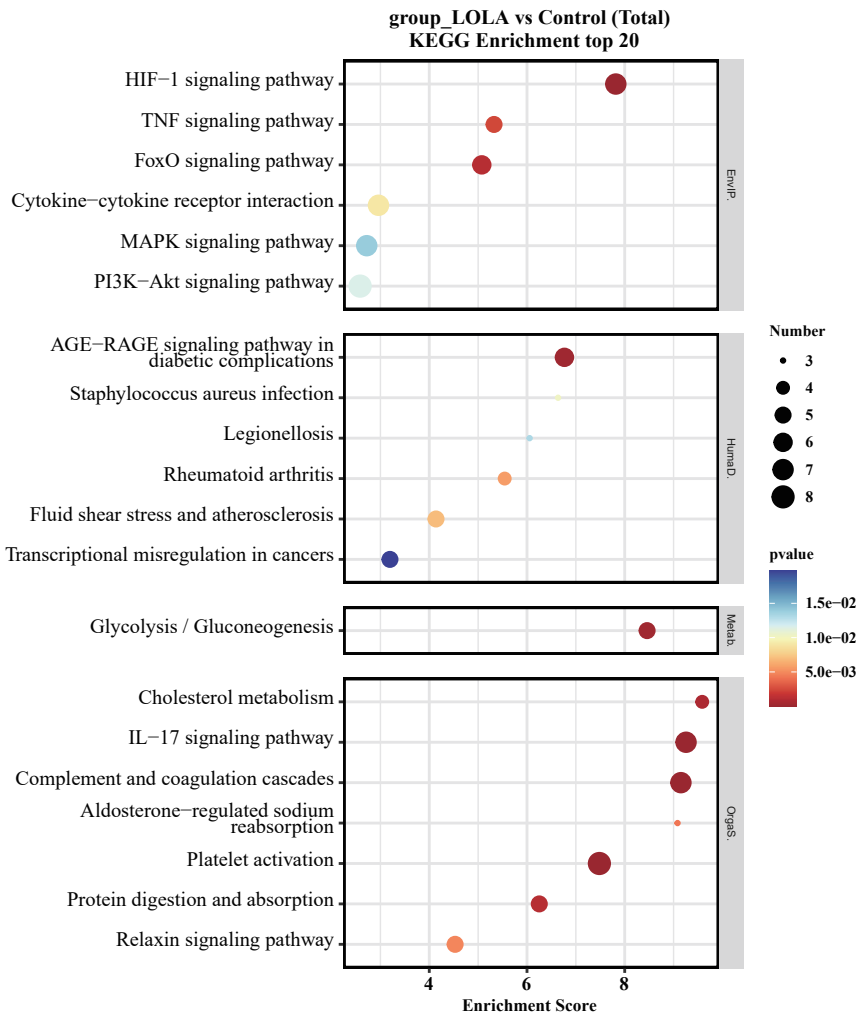

Supplement: Supplementary file 2 — Supporting File 2: advs75027‐sup‐0002‐FigureS1–S13.zip. [file ADVS-13-e21098-s001.zip › Figure S12.pdf]

Figure S13

A

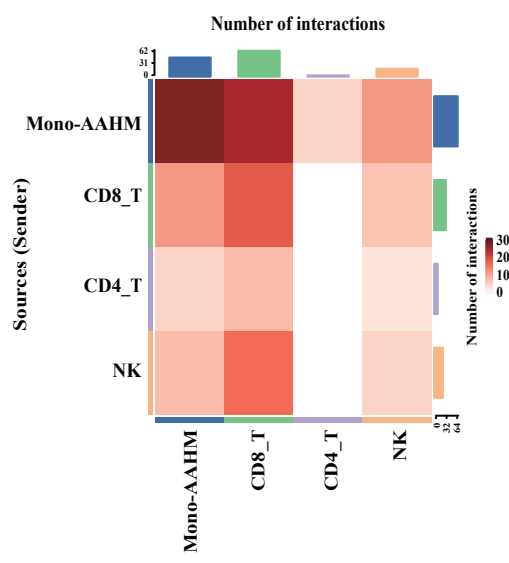

B

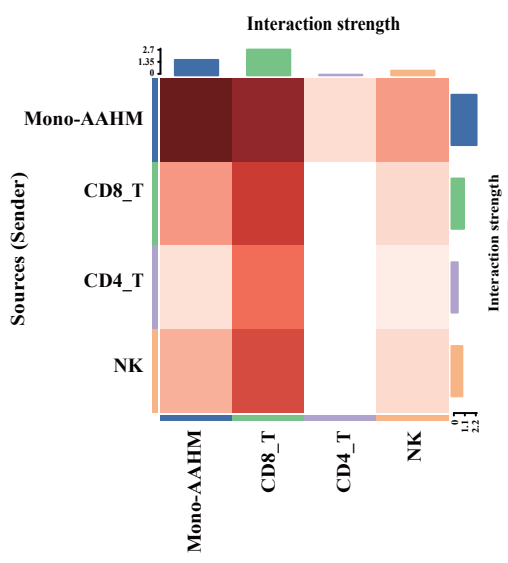

C

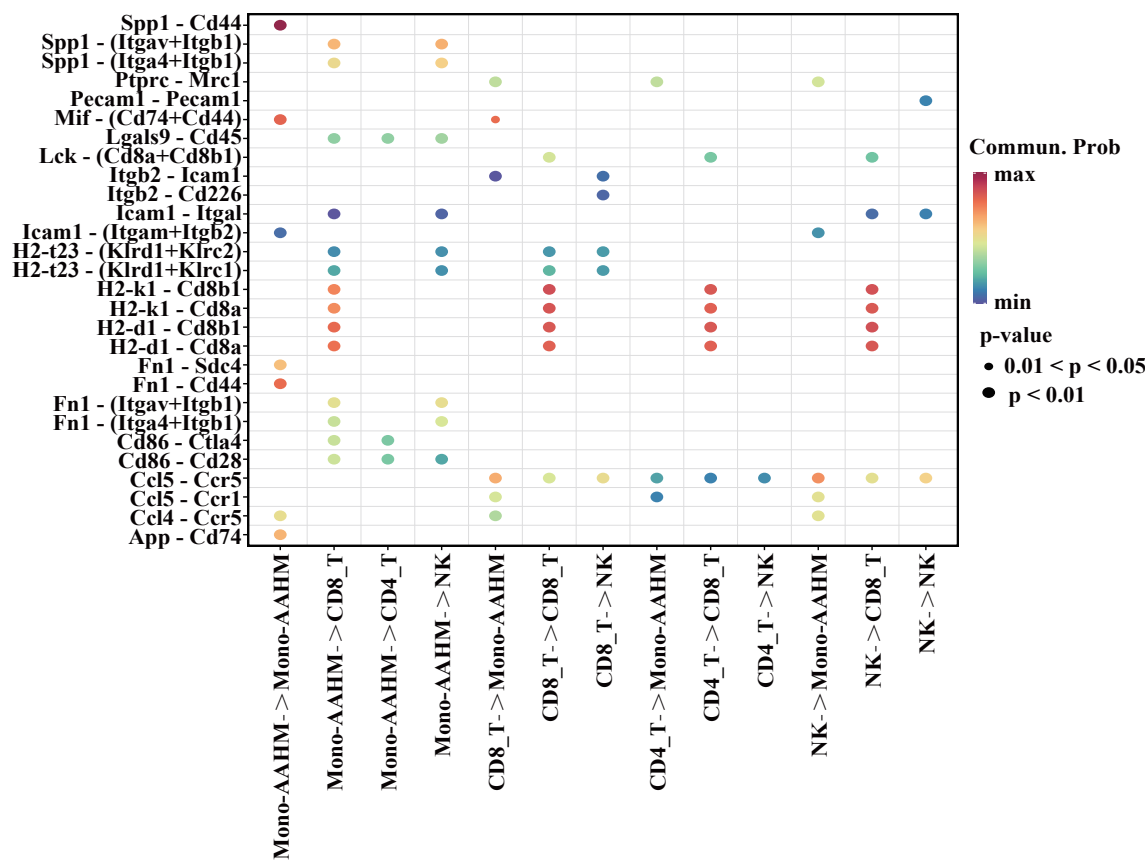

Supplement: Supplementary file 2 — Supporting File 2: advs75027‐sup‐0002‐FigureS1–S13.zip. [file ADVS-13-e21098-s001.zip › Figure S13.pdf]

Figure S2

A

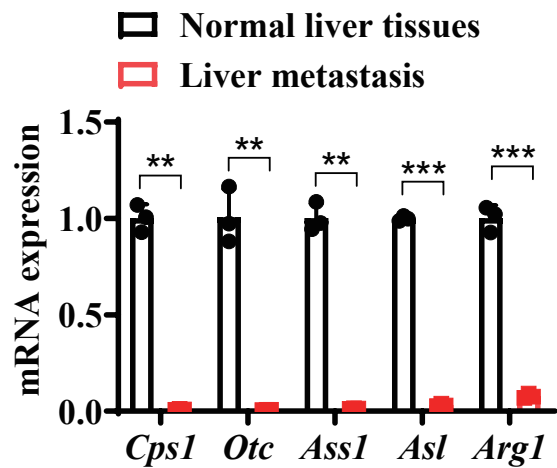

B

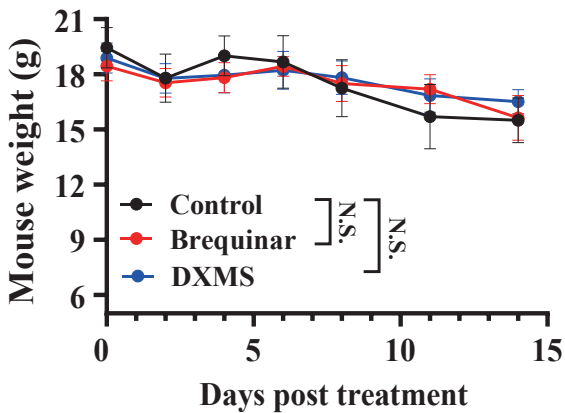

C

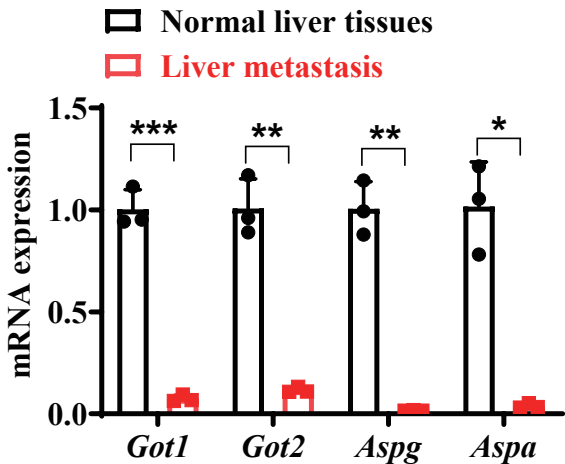

Supplement: Supplementary file 2 — Supporting File 2: advs75027‐sup‐0002‐FigureS1–S13.zip. [file ADVS-13-e21098-s001.zip › Figure S2.pdf]

**Figure S3**

**A**

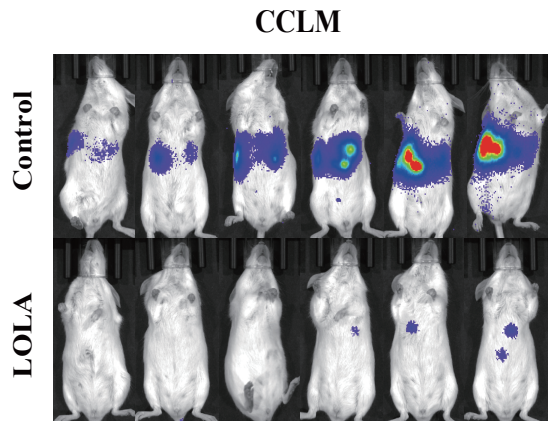

**B**

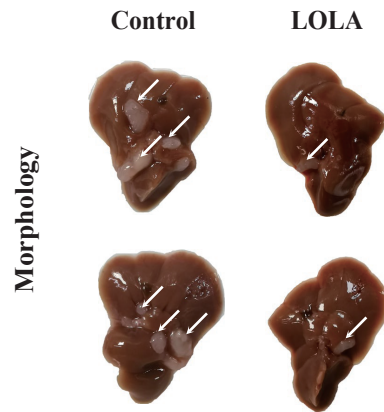

Supplement: Supplementary file 2 — Supporting File 2: advs75027‐sup‐0002‐FigureS1–S13.zip. [file ADVS-13-e21098-s001.zip › Figure S3.pdf]

Figure S4

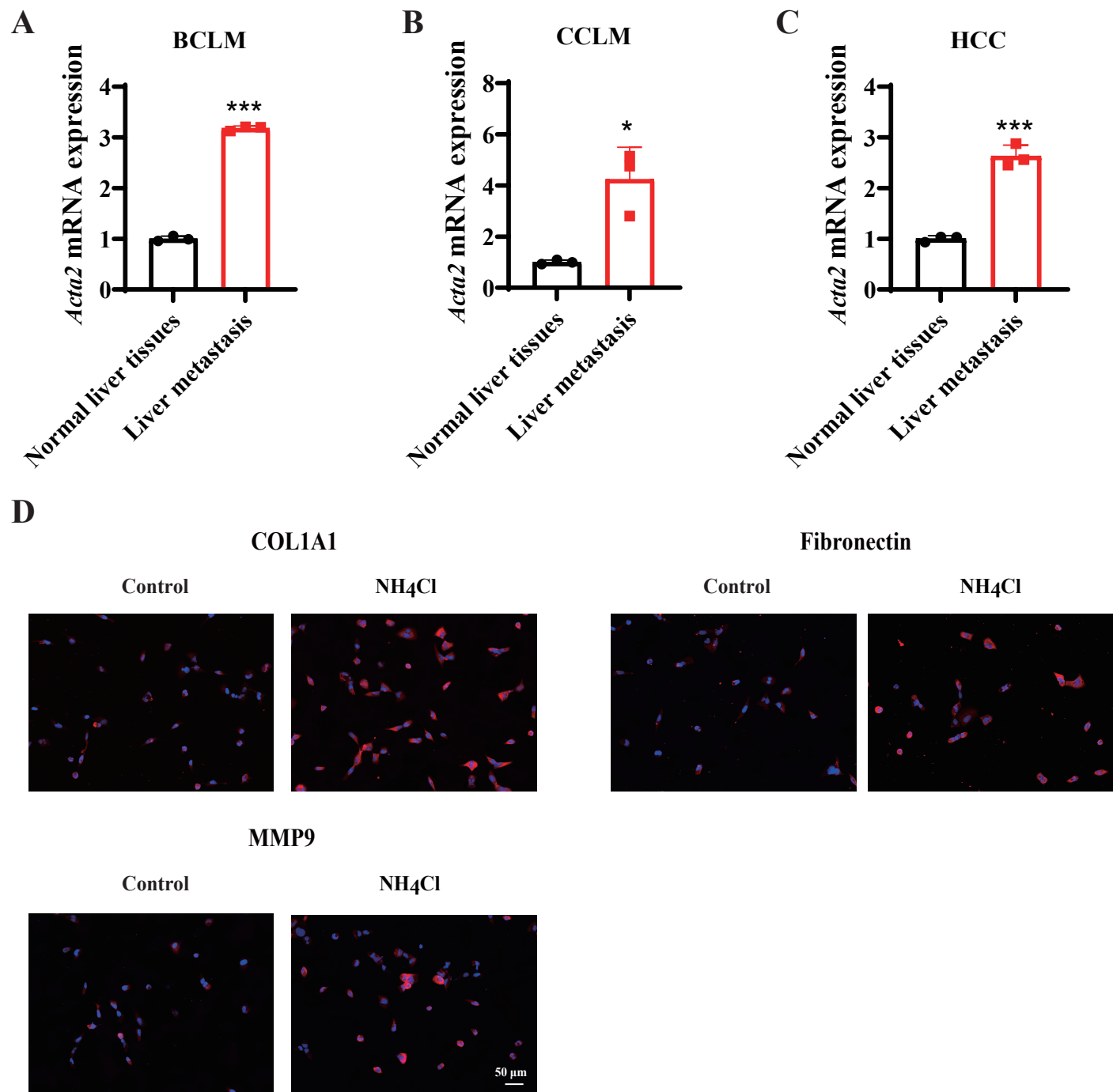

Supplement: Supplementary file 2 — Supporting File 2: advs75027‐sup‐0002‐FigureS1–S13.zip. [file ADVS-13-e21098-s001.zip › Figure S4.pdf]

**Figure S5**

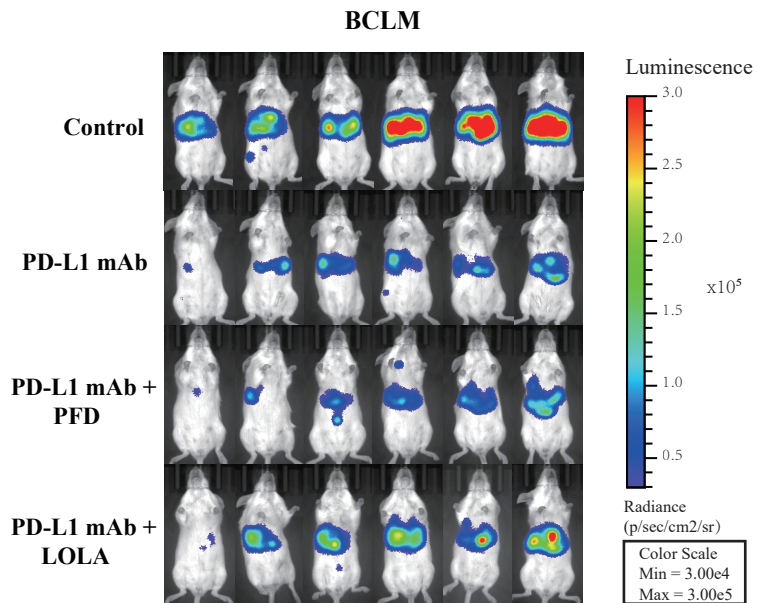

Supplement: Supplementary file 2 — Supporting File 2: advs75027‐sup‐0002‐FigureS1–S13.zip. [file ADVS-13-e21098-s001.zip › Figure S5.pdf]

Figure S6

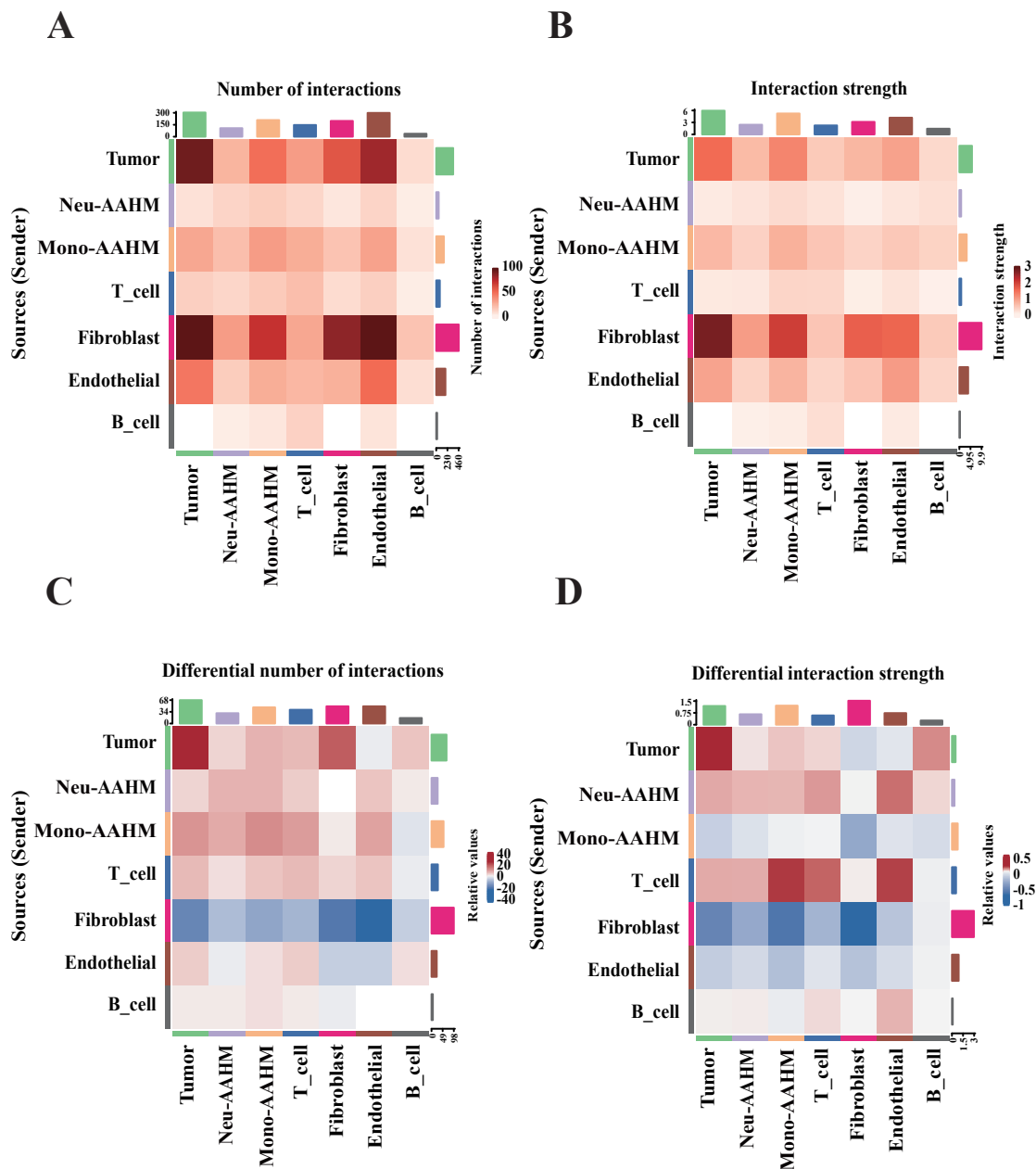

Supplement: Supplementary file 2 — Supporting File 2: advs75027‐sup‐0002‐FigureS1–S13.zip. [file ADVS-13-e21098-s001.zip › Figure S6.pdf]

Figure S7

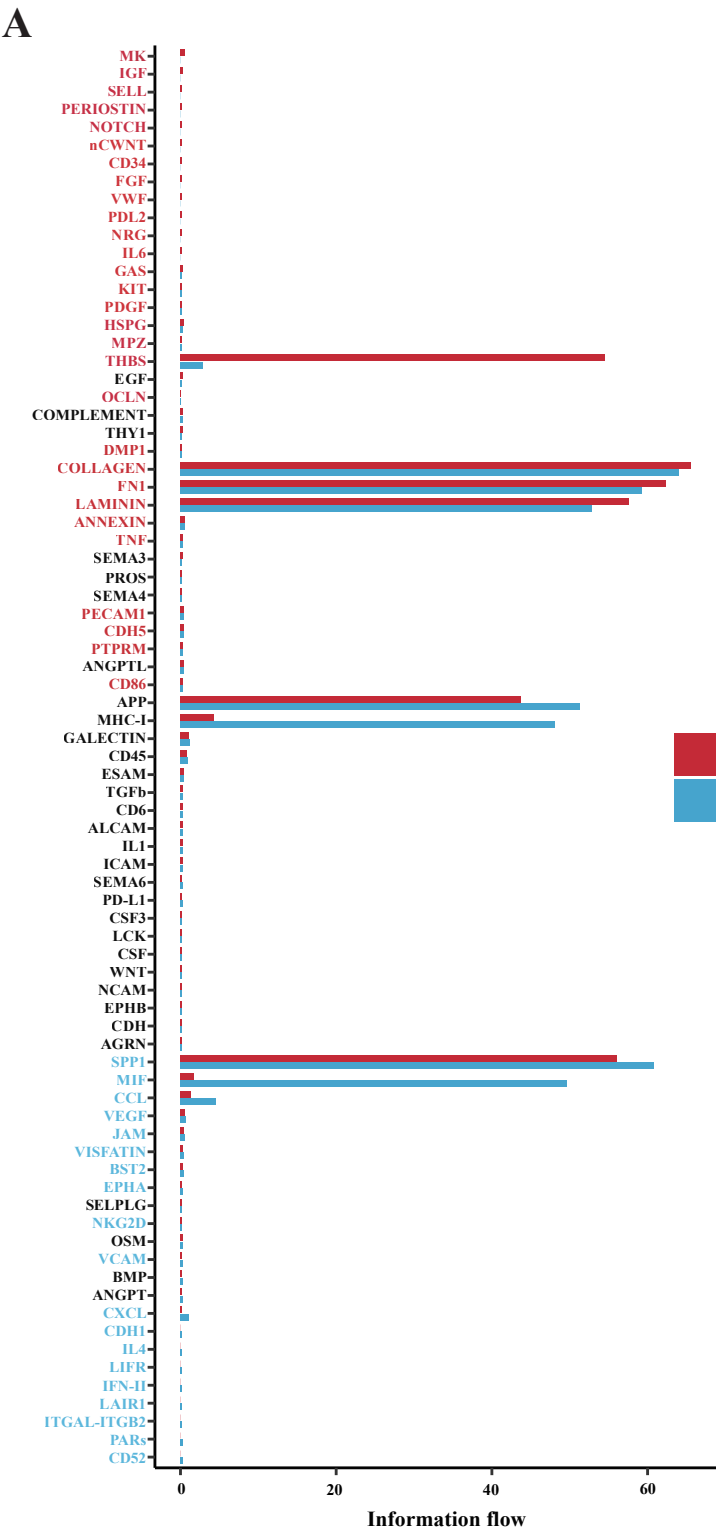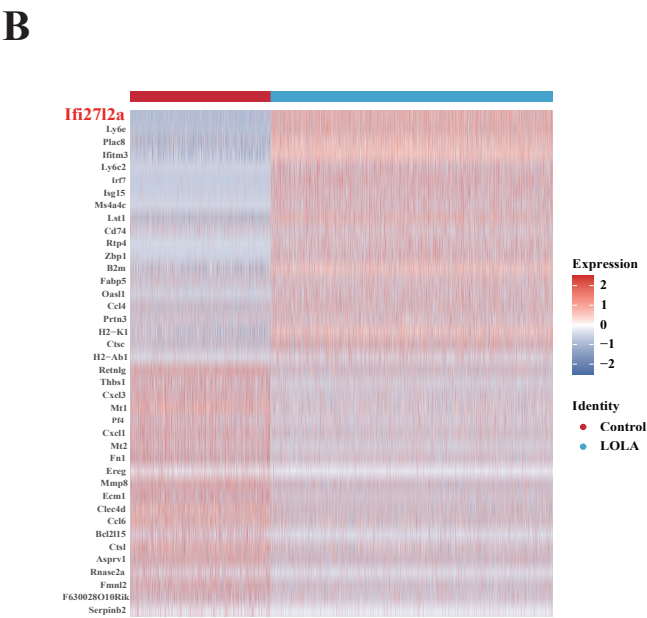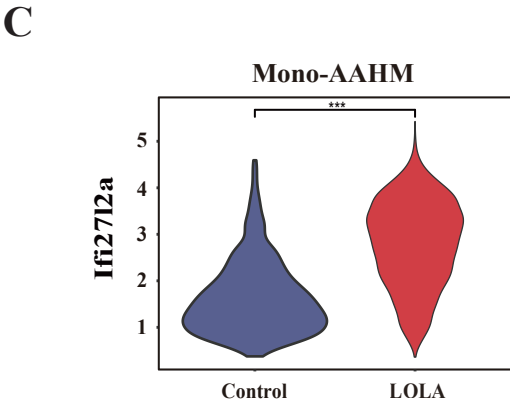

Supplement: Supplementary file 2 — Supporting File 2: advs75027‐sup‐0002‐FigureS1–S13.zip. [file ADVS-13-e21098-s001.zip › Figure S7.pdf]

Figure S8

A

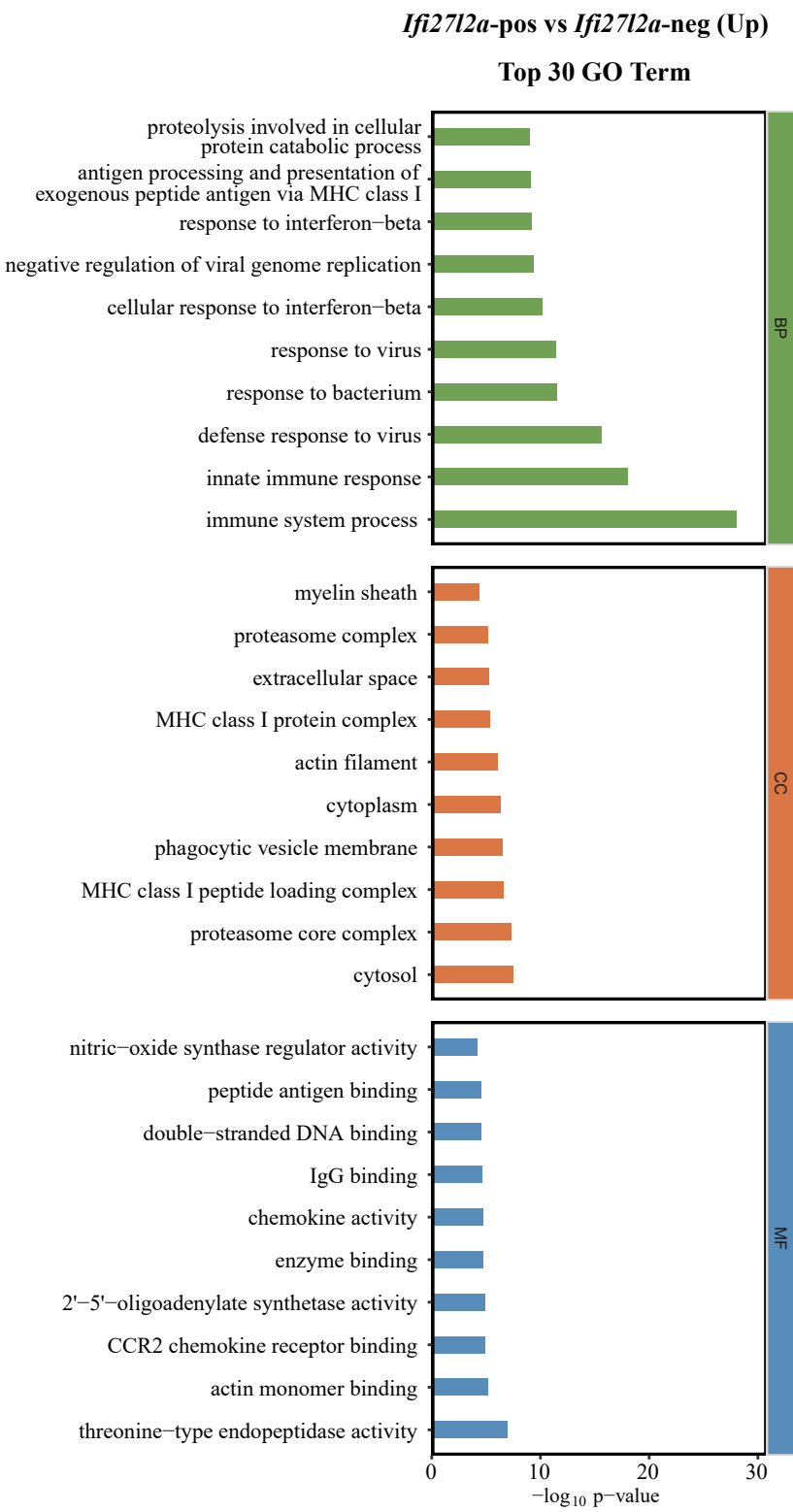

B

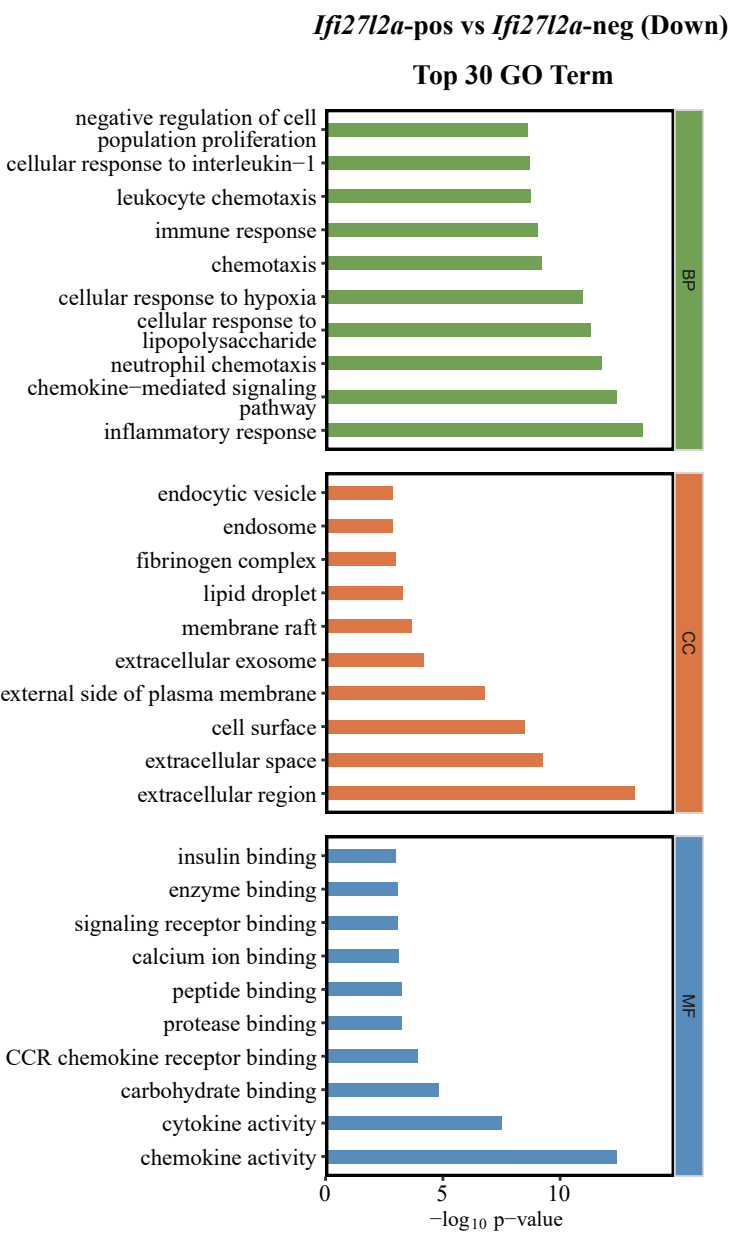

Supplement: Supplementary file 2 — Supporting File 2: advs75027‐sup‐0002‐FigureS1–S13.zip. [file ADVS-13-e21098-s001.zip › Figure S8.pdf]

Figure S9

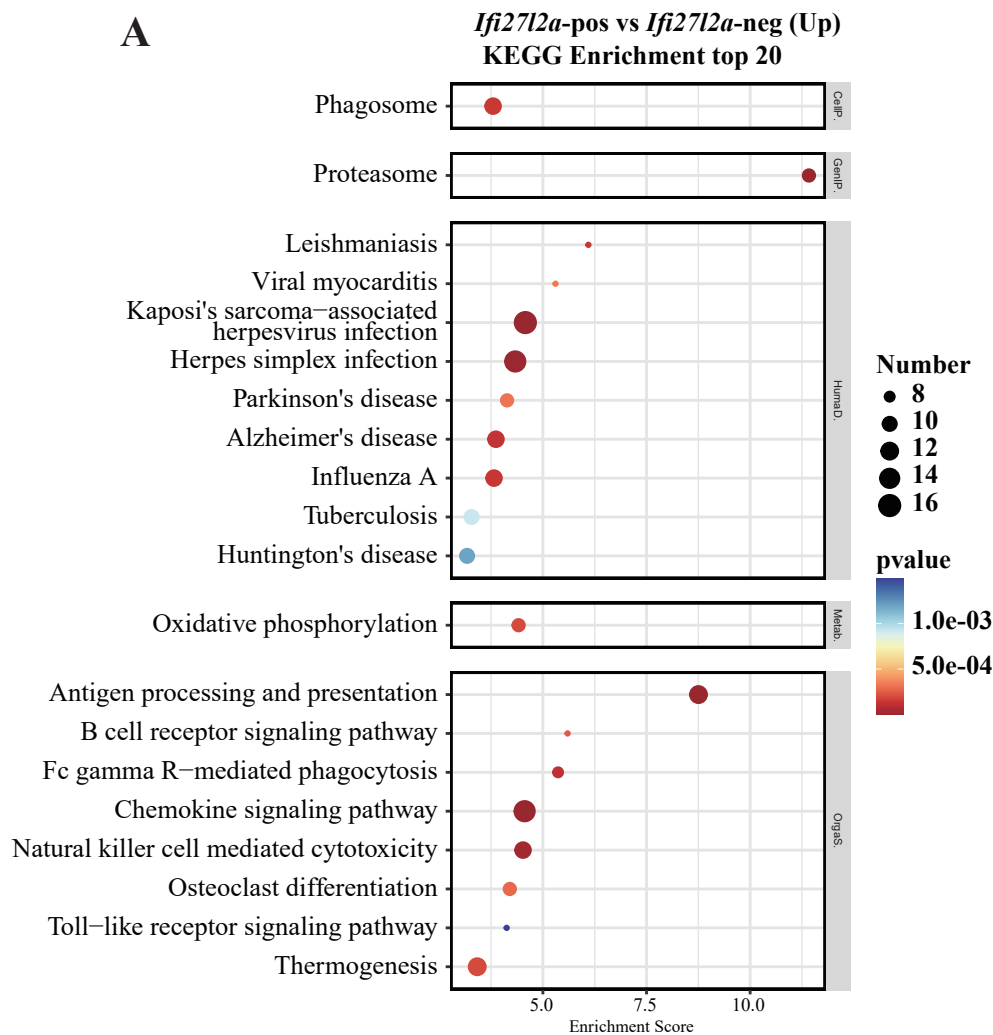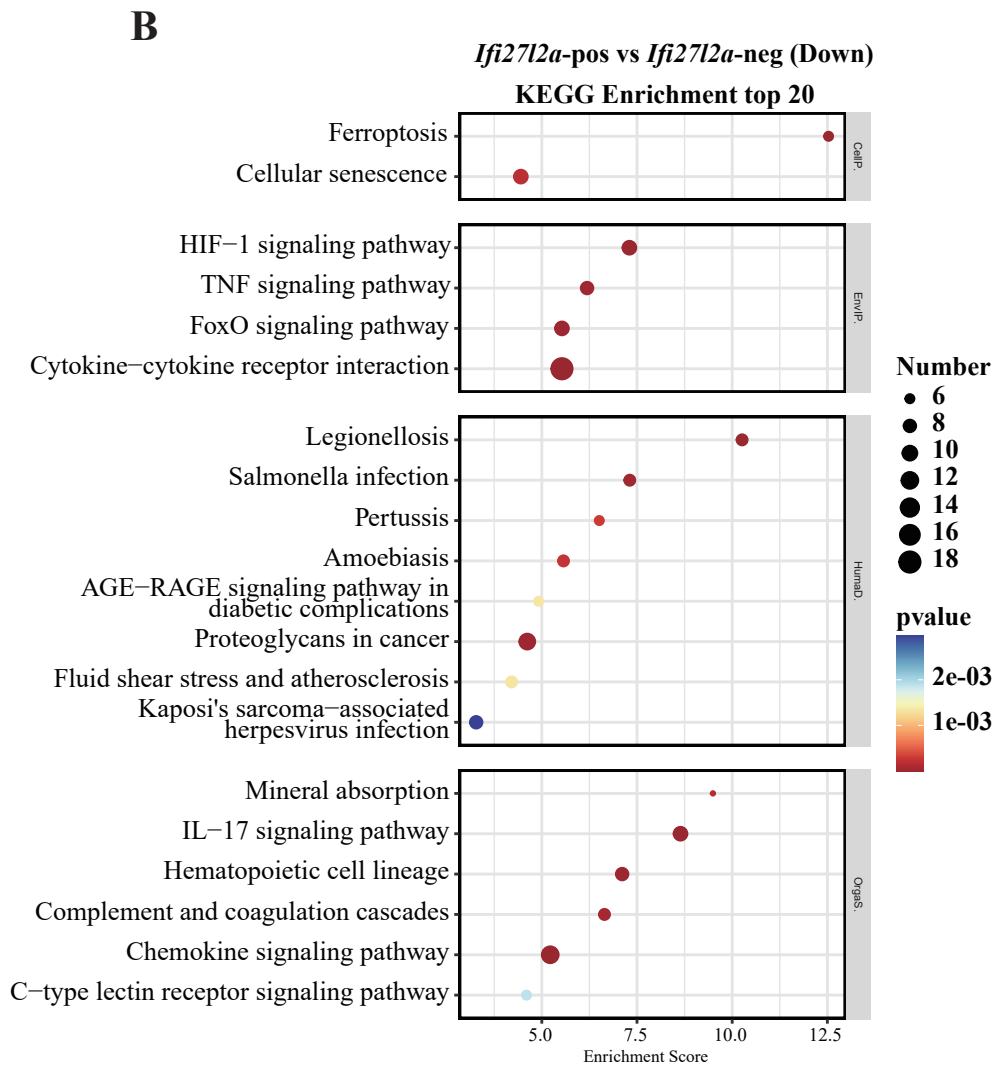

Supplement: Supplementary file 2 — Supporting File 2: advs75027‐sup‐0002‐FigureS1–S13.zip. [file ADVS-13-e21098-s001.zip › Figure S9.pdf]
